# Differential Gene Expression in Human Hippocampus With Aging

**DOI:** 10.1111/acel.70459

**Published:** 2026-03-26

**Authors:** Ander Saenz‐Antoñanzas, Manuel Moreno‐Valladares, Maider Muñoz‐Culla, Sara Cruces‐Salguero, Jon Landa, Ainhoa Alberro, Jhonatan Vergara‐Arce, Marta Arroyo‐Izaga, David Otaegui, Ander Matheu

**Affiliations:** ^1^ Group of Cellular Oncology, Biogipuzkoa (Biodonostia) Health Research Institute San Sebastian Spain; ^2^ Pathology Department, Donostia University Hospital San Sebastian Spain; ^3^ Neuroimmunology Group, Biogipuzkoa (Biodonostia) Health Research Institute San Sebastian Spain; ^4^ CIBER of Neurodegenerative Diseases (CIBERNED), Carlos III Institute Madrid Spain; ^5^ Department of Basic Psychological Processes and Their Development, School of Psychology University of the Basque Country (UPV/EHU) San Sebastian Spain; ^6^ BIOMICs Research Group, Microfluidics & BIOMICs Cluster, Department of Pharmacy and Food Sciences, Lascaray Research Center, University of the Basque Country (UPV/EHU) Vitoria Spain; ^7^ CIBER of Frailty and Healthy Aging (CIBERFES), Carlos III Institute Madrid Spain; ^8^ IKERBASQUE Basque Foundation for Science Bilbao Spain

**Keywords:** brain aging, hippocampus, RAD23B, transcriptome

## Abstract

Brain aging consists of a progressive loss of functional capacities, which is associated with a progressive cognitive decline and can lead to neurodegenerative diseases. Studies comparing the underlying molecular mechanisms of the human hippocampus between young and older adults remain scarce. In our study, we completed a transcriptomic analysis from hippocampal samples of different ages and performed 2 complementary analyses. A comparison between young and old groups revealed a set of genes differentially expressed in aged individuals linked to inflammation and immune system pathways, DNA repair, metabolism, or neural activity. Correlation analysis showed that the expression of an additional subset of 6 genes was associated with chronological aging. Among them, further analysis identified *RAD23B* as the most significant gene with a negative correlation of its mRNA and protein expression with age in the human hippocampus. Its expression was even lower in patients with Alzheimer's disease. RAD23B was mostly expressed in neurons and astrocytes, where studies in human primary cultures uncovered that it is required for cell survival and function. In summary, these results unravel dynamic gene expression changes that distinguish young from older adults and identify RAD23B as a putative biomarker and regulator of cell aging in the brain.

## Graphical abstract

2 complementary analysis from a transcriptome study in human hippocampal samples of different ages revealed a set of genes differentially expressed in aged individuals linked to inflammation and immune system pathways, DNA repair (RAD23) or neural activity.

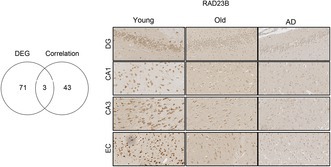

## 1 Introduction

Aging is a systemic and multifactorial process characterized by the decline of physical and mental capacities that occurs due to the gradual accumulation of cellular damage (Lopez‐Otin et al. 2023). Aging affects the entire organism in humans; however, brain aging is especially distinctive since the brain is the most structurally and functionally complex organ. Thus, aging promotes changes to the brain size, structure, vasculature, biology, and impacts on cognitive abilities (Murman 2015). Cognitive abilities such as memory, thinking, concentration, movement, emotions, and other functions deteriorate with aging. Aging is also the major risk factor for the onset of neurodegenerative diseases, being Alzheimer's disease (AD) the most common form of dementia in older adults (Bishop et al. 2010).

The hippocampus is a region of the temporal lobe, which contains the dentate gyrus (DG) and the cornu ammonis (CA), divided into CA1–CA4 regions, each of which includes distinct laminar organization and specialized cell types. The hippocampus supports several relevant biological processes and it is responsible for learning, memory, navigation, stress response, and emotion (Lisman et al. 2017).

The hippocampus is a vulnerable region that, with aging, suffers structural, functional, cellular and molecular changes that collectively contribute to cognitive decline (Aimone et al. 2014) and the onset of neuropsychiatric and neurodegenerative diseases including AD (Small et al. 2011). Although the structural, functional and cellular changes are relatively well known, the molecular ones and their relationship with the others are an area of intense research currently. Structural alterations include reduction in hippocampal volume, while functionally, the aging hippocampus exhibits alterations in neural activity and connectivity and impaired synaptic plasticity (Fjell and Walhovd 2010). At cellular level, aging promotes significant changes in the different cell types. Neurons exhibit the loss of nerve fibers and demyelination, reduced dendritic branching and spine density, vulnerability to oxidative stress, and accumulation of toxic proteins and damage (Isaev et al. 2018). Besides neurons, aging also affects glial cells, which lose their ability to maintain normal brain homeostasis, including correct neurotransmission, neural damage repair, metabolism or cerebral blood flow (Soreq et al. 2017). In particular, astrocytes become reactive, releasing pro‐inflammatory cytokines that contribute to neuroinflammation and neuronal damage. Microglia also become activated with age, while oligodendrocyte function and myelin production decline (Soreq et al. 2017; Navarro Negredo et al. 2020).

At molecular levels, the study of brain aging has drawn the attention of the scientific community in the research of those aging‐associated mechanisms underlying brain dysfunction (Ham and Lee 2020; Higgins‐Chen et al. 2021; Lu et al. 2004). Advances in high‐throughput “omic” technologies are advancing our understanding of how aging reshapes the molecular landscape of the human hippocampus, enabling comprehensive profiling of biological and molecular modifications. Among them, transcriptomic approaches have revealed widespread gene expression changes in the aging hippocampus, with different studies showing altered expression of genes involved in processes such as genomic instability and DNA repair, synaptic transmission and intercellular communication, mitochondrial function, inflammatory pathways, and stress responses (Ham and Lee 2020; Mattson and Arumugam 2018). More recently, single‐cell RNA sequencing and spatial transcriptomics have further extended this characterization allowing cell‐type‐specific resolution of transcriptional changes and enabling mapping of gene expression changes within the different hippocampal subregions (Wu et al. 2025; Wang et al. 2022; Su et al. 2023; Zhou et al. 2022; Matheu et al. 2005). Previous studies have demonstrated that alterations in gene expression profiles can precede histopathological degeneration of the brain during aging and underlie age‐associated cognitive declines at the molecular level (Ham and Lee 2020). In line with these findings, we have recently characterized the transcriptome of the hippocampus of centenarians and compared it to young and old groups identifying a specific molecular pattern in centenarians' brain (Saenz‐Antonanzas et al. 2024). Therefore, understanding the changes that occur with aging in the human hippocampus is important in order to understand aging‐associated cognitive decline and disease onset.

In order to further study the impact of aging in human brain with special emphasis in the hippocampus, in the present study we performed a transcriptomic analysis in hippocampus samples derived from individuals of different ages finding a differential expression pattern in old compared to young individuals, which was related to previously described biological processes such as inflammation and immune system pathways, synaptic activity, DNA repair and metabolism and linked to different cell types. An additional analysis identified the molecular mechanisms associated with chronological aging, revealing changes in the expression of several genes that correlated positively or negatively with age and were also altered in AD.

## 2 Material and Methods

### 2.1 Human Brain Samples and Public Available Datasets

For transcriptome and quantitative real‐time polymerase chain reaction (qRT‐PCR) studies, the BIOMICs group provided the human brain samples (cohort 1), obtained from forensic autopsy, which are part of the collection C.0000217 from Instituto Salud Carlos III Biobank register (https://biobancos.isciii.es/ListadoColecciones.aspx). The transcriptomic study was performed in coronal sections of human hippocampal samples from 16 individuals including young individuals (*n* = 5, 27–49 years old) and old (*n* = 11, 58–100 years). The samples included 5 cuts of 3 cm thick each from frontal to occipital area. The DEG analysis was completed with samples 1 to 13 and the correlation with the 16 (Table S1). q‐RTPCR validation was completed in additional samples of up to 120 individuals from the same cohort 1 including young (*n* = 78, 27–50 years old) and old (*n* = 42, 65–96 years old) (Table S2) (Carrasco‐Garcia et al. 2019). These brains were kept in RNA later and frozen in liquid nitrogen.

Immunohistochemistry (IHC) studies were completed in paraffin sections from samples obtained from Donostia Hospital (cohort 2) (Table S3) of 26 individuals including young (*n* = 6, 28–47 years), old (*n* = 12, 58–90) and an additional group of neurodegenerative—AD (*n* = 8, 62–82 years) (Moreno‐Valladares et al. 2020). Brains were kept in a fixative solution (4% paraformaldehyde) for a period of not less than 24 h. Old individuals included in the study showed absence of AD diagnosis as well as the lack of neuropathological injuries in the regions analyzed. AD cases were diagnosed in the Neurology and Pathology Services of Donostia Hospital. Postmortem interval (PMI) was limited to 12 h due to its effects on brain proteins. Single‐cell RNA‐Seq data was analyzed from “The Human Protein Atlas” (http://www.proteinatlas.org) using the consensus dataset consisting of the normalized expression (nTPM) levels. An additional RNA‐Seq of cell types isolated from human brain was retrieved from “Brain RNA‐Seq” (https://brainrnaseq.org/) (Zhang et al. 2016). RNA‐Seq data from old individuals were obtained from “Aging, Dementia and Traumatic Brain Injury (TBI) Study” (https://aging.brain‐map.org/rnaseq/search), encompassing 54 participants (female *n* = 22, male *n* = 32) from 78 to 100+ years old, among whom 24 presented dementia. Additional transcriptomic data of old and young individuals was retrieved from GSE53890 (Lu et al. 2014), from the Adult Genotype Tissue Expression (GTEx) project (Consortium GT 2020) and from the Human Brain Transcriptome (https://hbatlas.org/). Data from Alzheimer Disease Progression Atlas were obtained in https://ad‐progression‐atlas.partners.org/?page=home (Serrano‐Pozo et al. 2024).

### 2.2 Mouse Studies

The C57BL/6J mouse strain was obtained from The Jackson Laboratory and housed in pathogen‐free barrier areas of the Biodonostia Health Research Institute. For brain extraction, mice were anesthetized with isoflurane and culled by decapitation. In situ hybridization and expression data were obtained from Allen Brain Atlas data portal (https://mouse.brain‐map.org/). Transcriptomic data of mouse NSCs from the subependymal zone and subventricular zone were retrieved from GSE138243 (Belenguer et al. 2021) and GSE168189 (Marques‐Torrejon et al. 2021), respectively. An additional single‐cell RNA‐Seq from mouse hippocampus with AD was obtained from GSE141044 (Zhong et al. 2020) in the scREAD database (Jiang et al. 2020).

### 2.3 Transcriptome Analysis

Gene expression array was performed from 5 ng of RNA using *Clariom S array* (902922. Thermo‐Fisher Scientific, Waltham, MA, USA) following manufacturers' instructions, which measure gene‐level expression from > 20.000 well‐annotated genes. Data were analyzed with Transcriptome Analysis Console (TAC) software v4.0. Data were normalized using the Robust Multi‐array Average (RMA) and batch effect was eliminated using batch effect module of the TAC software. Then, studied groups were compared with Limma differential expression analysis to find differentially expressed genes with *p* < 0.05 and fold change ≥ |2| were selected. Gene Ontology (GO) analysis was performed using PANTHER GO‐Slim Biological Process (http://www.pantherdb.org/panther/goSlim.jsp). Additionally, correlation was calculated between the expression and age of the individual for each transcript. The expression of 46 genes was found to be significantly correlated (*p* < 0.001), 6 of them having a correlation coefficient higher than |0.8|.

### 2.4 Primary Astrocyte Cultures

Normal Human Astrocytes (1800, ScienCell, California, CA, USA) were cultured in adhesion in culture plates pre‐treated with 15 μg/mL poly‐L‐lysine. Astrocyte medium kit (1801, ScienCell) was employed and cells were maintained in culture conditions at 37°C, 95% humidity, 21% O_2_ and under 5% CO_2_ pressure. NHA were maintained in culture and passages were performed every 4–5 days (Saenz‐Antonanzas et al. 2024). The expression of genes was studied at early (Lopez‐Otin et al. 2023; Murman 2015; Bishop et al. 2010) and late (Wu et al. 2025; Wang et al. 2022; Su et al. 2023; Zhou et al. 2022) passages after total RNA extraction from cell cultures.

### 2.5 Lentiviral Infections

NHA were transduced with lentiviral vectors containing a plasmid with silencing sequence of RAD23B (*shRAD23B*, TRCN0000003955, Sigma‐Aldrich, St Louis, MO, USA). pLKO.1 puro (Addgene plasmid #8453) was used as control. Lentiviral infections were performed overnight with a multicity of infection (MOI) of 10 at 37°C and 5% CO_2_ in astrocyte medium. After 48 h, infected cells were selected in the presence of 2 μg/mL puromycin (Sigma‐Aldrich) for 48–72 h.

### 2.6 Cell Growth and Senescence Assays

NHA were plated in a density of 10^4^ cells in 6‐well plates and number of cells was determined at days 1, 4 and 8. Data were represented indicating the total number of cells per experimental condition in each time point. Senescence assay was performed using the Senescence β‐Galactosidase Staining Kit (9860, Cell Signaling, Danvers, MA, USA) according to the manufacturer's guidelines.

### 2.7 Cell and Tissue IF

Cell IF was performed following standard procedures as described in previous studies (Arrizabalaga et al. 2017). Cells were incubated with primary Ki67 (1:500, 15580, Abcam, Cambridge, UK) and Caspase 3 (1:500, AF835, Minneapolis, MN, USA) antibodies, followed by the respective secondary antibodies donkey anti‐mouse 488 (1:1000, A21202, Invitrogen, Carlsbad, CA, USA) or donkey anti‐rabbit 555 (1:1000, A31572, Invitrogen). For nuclear DNA staining, Hoechst 33,342 (Sigma‐Aldrich, Burlington, MA, USA) was used. Pictures were taken with an Eclipse 80i microscope and processed with the NIS Elements Advances Research software (Tokyo, Japan).

Tissue IF was performed in formalin fixed brain samples. Deparaffination protocol was performed by heating samples at 15′ at 65°C, followed by immersion in xylene and a series of alcohol solutions of decreasing concentration (100%, 70%, 50%, and distilled water), with each immersion lasting 5 min. Finally, samples were heated for 1 h in citrate buffer for antigen retrieval. The sections were permeabilized and blocked for 2 h with 2% normal donkey serum and they were incubated with anti‐RAD23B (1:300 H00005887‐B02P, Novus Biologicals), GFAP (1:300, 13‐0300, Invitrogen) and TUJ1 (1:500, 802001, Biolegend, San Diego, CA, USA) primary antibodies at 4°C overnight, followed by the respective secondary antibodies donkey anti‐rat 488 (1:1000, A21208, Invitrogen), donkey anti‐rabbit 488 (1:1000, A21206, Invitrogen) or donkey anti‐mouse 555 (1:1000, A31570, Invitrogen). Nuclear DNA was stained with DAPI (10 mg/mL, 40011, Biotium, Fremont, CA, USA). The preparation was mounted with ProlongTM Gold antifade mounting media (P36930, Invitrogen) and IF was evaluated with SP5 laser scanning confocal microscope (TCS SP5, Leica, Wetzlar, Germany). Processing and analysis were performed on the maximal intensity projection of the *z*‐stack using Fiji software. Quantification was measured based on protein positive signal respect to total nuclei in DAPI channel.

### 2.8 Immunohistochemistry (IHC)

5 μm sections were done using a microtome (HM355S, Thermo Scientific), deparaffined in xylene and rehydrated in a series of graded alcohols as described above. Heat‐induced antigen retrieval with citrate buffer was performed for 10 min. Endogenous peroxidase was blocked with 5% hydrogen peroxide in methanol for 15 min. After incubation with PBS 0.3%‐Triton X‐100 5% FBS for blocking, sections were incubated with the respective primary antibody, anti‐RAD23B (1:100, H00005887‐B02P, Novus Biologicals, Centennial, CO, USA), anti‐SMPD4 (1:100, HPA049426, Atlas antibodies, Stockholm, Sweden) or anti‐ANKRD18B (1:100, PA5‐61220, Thermo‐Fischer Scientific) at 37°C for 2 h. The sections were then washed and incubated with MACH 3 Rabbit Probe and MACH 3 Rabbit HRP‐Polymer (M3R531, Biocare Medical, Pacheco, CA, USA). Color was developed with 3,3′‐Diaminobenzidine (DAB) and nuclei were counterstained with hematoxylin. Sections were visualized with a light microscope and scanned with Virtuoso v.5.6.1 software (Roche, Basel, Switzerland).

### 2.9 RNA Extraction and Analysis by q RT‐PCR

Total RNA extraction was performed using Trizol (11578616, Life Technologies, Carlsbad, CA, USA). Reverse transcription (RT) was performed using the Maxima First Strand cDNA Synthesis Kit (K1641, ThermoFisher), following the manufacturer's instructions. To analyze gene expression, quantitative real‐time polymerase chain reaction (qRT‐PCR) with 20 ng of cDNA was performed by Absolute SYBR Green mix (10209284, Thermo Scientific) on a CFX384 thermal cycler (Bio‐Rad, Hercules, CA, USA). Transcript levels were normalized to Glyceraldehyde‐3‐phosphate dehydrogenase (GAPDH) and measured using the ^ΔΔ^Ct relative quantification method.

### 2.10 Western Blot Analysis

Immunoblots were performed following procedures previously described (Matheu et al. 2005). Specific antibodies against RAD23B (1:250, H00005887‐B02P, Novus Biologicals) and β‐actin (1/100000, A5441, Sigma‐Aldrich) were used in the study, followed by Horse anti‐mouse HRP 1/1000, 7076S, Cell Signaling. Detection was performed by chemiluminescence using NOVEX ECL Chemi Substrate and SuperSignal West Femto Maximum Sensitivity Substrate (34095, ThermoFisher).

### 2.11 Data Analysis

Statistical analyses and graphics were performed using Microsoft Office Excel, IBM SPSS Statistics 20, R Studio and GraphPad Prism 8 software. Data are represented as mean values ± SEM, with the number of experiments (*n*) carried out for each assay. Unless otherwise indicated, statistical significance was calculated by Student's *t*‐test ^≠^ *p* < 0.1, *p ≤ 0.05; **p ≤ 0.01; ***p ≤ 0.001. In case of multiple tests, we included the results with multiple testing correction by False Discovery Rate (FDR). For correlation analysis, we first performed a Kolmogorov–Smirnov test for the assessment of normality and then we used Pearson's coefficient when samples were normally distributed or Spearman's coefficient when they were not normally distributed. Public available transcriptomic data was analyzed following the default limma pipeline in R for differential expression analysis, and the fold change was represented. Single‐cell RNA‐Seq was loaded into Seurat, normalized, and represented through violin plots in R.

### 2.12 Ethics Approval

This study was approved by the Clinical Research Ethics Committee of the DH (AMM‐MHP‐2019‐1) and adhered to the tenets of the Declaration of Helsinki by the World Medical Association regarding human experimentation. Animal studies were approved by the Biodonostia Institute Animal Care and Use Committee in accordance with the Spanish Royal Decree 53/2013.

## 3 Results

### 3.1 Transcriptome Analyses Reveal Differentially Expressed Genes With Age in the Hippocampus

**FIGURE 1:**
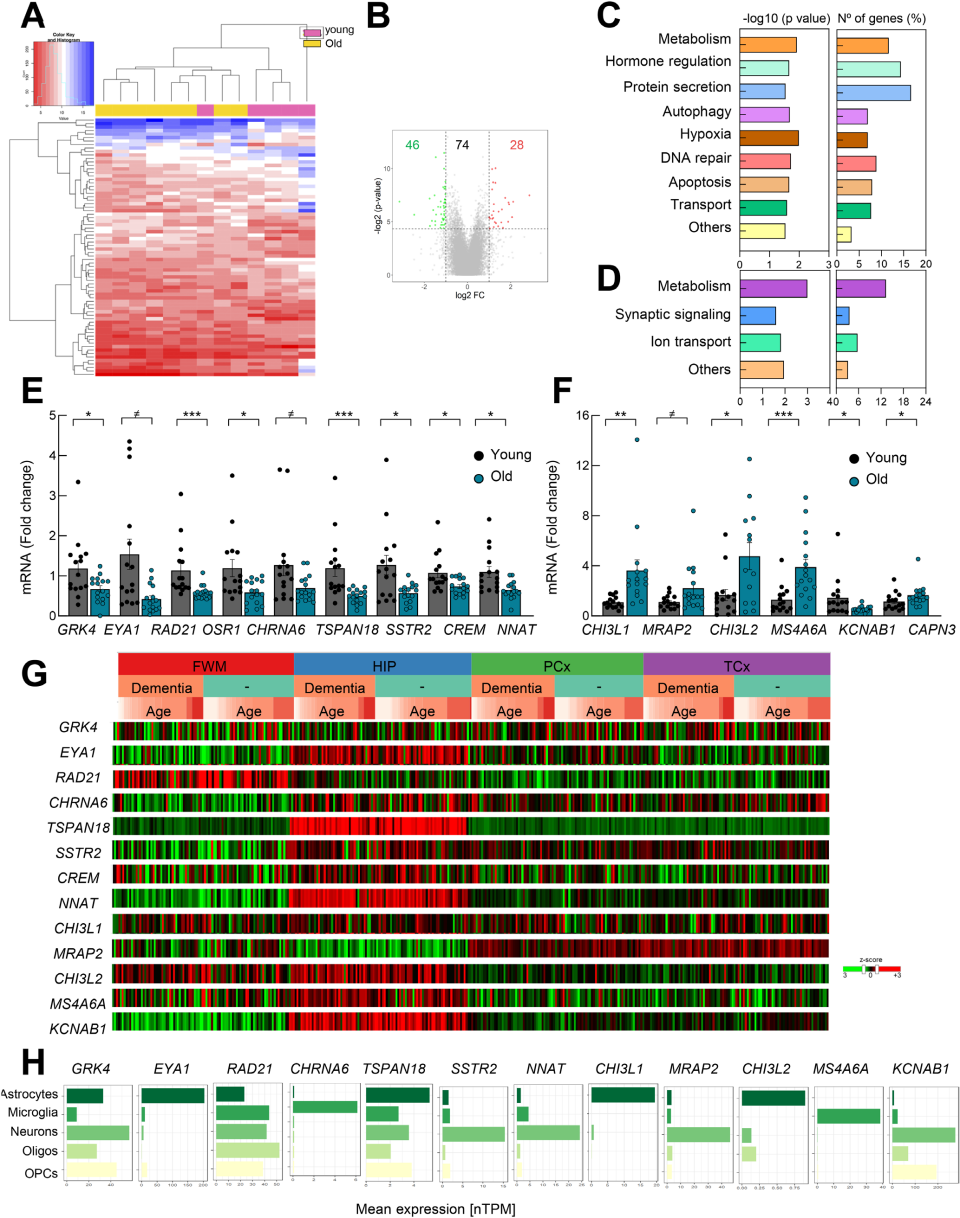
Transcriptome analyses reveal differentially expressed genes with age in the hippocampus. (A) Volcano plot of decreased (green) and increased (red) genes in the analysis of old (*n* = 8) versus young (*n* = 5) samples. All genes selected *p*‐value < 0.05 and FC ≥ |2|. (B) Number of genes upregulated and downregulated in old group. (C, D) Representative bar plots of biological processes associated with (C) decreased or (D) increased genes after Gene Ontology (GO) analysis. (E, F) mRNA expression levels of selected genes by qRT‐PCR in young (*n* = 15) and old individuals (*n* = 16). (G) Expression of genes in white matter of forebrain (FWM), hippocampus (HIP), parietal neocortex (PCx) and temporal neocortex (TCx) of older individuals (*n* = 30 healthy, *n* = 24 dementia) from https://aging.brain‐map.org. (H) Normalized expression (nTPM) of identified genes from Single cell RNAseq data from http://www.proteinatlas.org. The statistical significance was (^≠^ *p* < 0.1, *p < 0.05, **p < 0.01, ***p < 0.001).

We performed transcriptome study of human hippocampus samples from individuals of different ages divided in young vs. old, and we observed a differential gene expression pattern between the two groups (Figure 1A). In particular, 74 genes were differentially expressed with *p*‐value < 0.05 and FC ≥ |2|, among which 46 were decreased and 28 increased in older individuals compared to young ones (Figure 1B, Table S4). GO analysis of significantly altered genes between the two groups revealed that the top canonical biological processes of downregulated genes were associated with metabolism, DNA repair, hormone regulation, protein secretion, autophagy, hypoxia, apoptosis, and transport (Figure 1C). On the contrary, processes associated with metabolism, synaptic signaling and ion transport were detected among upregulated genes (Figure 1D). In order to confirm these results, we measured the mRNA expression of a selection of identified genes by q‐RTPCR in the cohort of 33 individuals that were at the extremes including young (*n* = 16, 27–45 years) and old (*n* = 17, 76–96 years). In particular, 33 genes (18 decreased and 15 increased in the transcriptome) were selected based on the FC, *p*‐values and the pathways in which they participate. Among them, we detected a statistically significant decreased expression of genes linked to metabolism or transport pathways (*GRK4*, *TSPAN18*, *SSTR2*, *PPP1R1B*), DNA repair (*EYA1*, *RAD21*, *ZBTB10*), development (*OSR1*, *CREM*, *ZIC1*, *EFEMP2*, *NNAT*) and neurotransmitter release or activity (*CHRNA6*) (Figures 1E and S1). Moreover, we found statistically significant increased levels of genes mainly linked to inflammation or immune system pathways (*CHI3L1*, *CHI3L2*, *SERPINA3*, *CERCAM*) and metabolism (*MRAP2*, *CAPN3*, *PD1E1C*), transport (*ANKRD30*, *MS4A6A*) and synaptic activity (*DLGAP1*, *KCNAB1*) (Figure 1F). No differences were detected when the samples were divided by sexes (Figure S2A–D). These results reveal a set of differentially expressed genes with age.

Taking advantage of data from public available datasets we characterized the expression of the set of identified genes in different brain regions and cell types. First, using data obtained from TBI study (https://aging.brain‐map.org/rnaseq/search), we observed that the expression of most genes (*EYA1*, *TSPAN18*, *SSTR2*, *CREM*, *NNAT*, *CHI3L2*, *MS4A6A*, *KCNAB1*) was higher in the hippocampus compared to additional brain regions such as white matter of the forebrain, parietal and temporal neocortex (Figure 1G). In addition, their expression was also high in cases with dementia (Figure 1G). We also studied the expression of the ortholog genes in adult C57BL6 mice brain sections using “Allen Mouse Brain Atlas” (https://mouse.brain‐map.org/). In this context, most genes were also enriched in the DG (Figure S3).

Next, we studied their expression in different brain cell types. For this, we analyzed single‐cell RNA‐Seq studies performed in human brains from The Human Protein Atlas (http://www.proteinatlas.org) and from an additional study (http://www.brainrnaseq.org) (Zhang et al. 2016). We noticed that *EYA1*, *CHI3L1*, *CHI3L2*, *MS4A6A*, and *RAD21* appeared to be expressed mostly by glial cells. In contrast, *CHRNA6*, *SSTR2*, *NNAT*, and *MRAP2* were expressed mostly by neurons (Figures 1H and S4).

### 3.2 The Expression of 6 Genes Correlates With Chronological Aging

**FIGURE 2:**
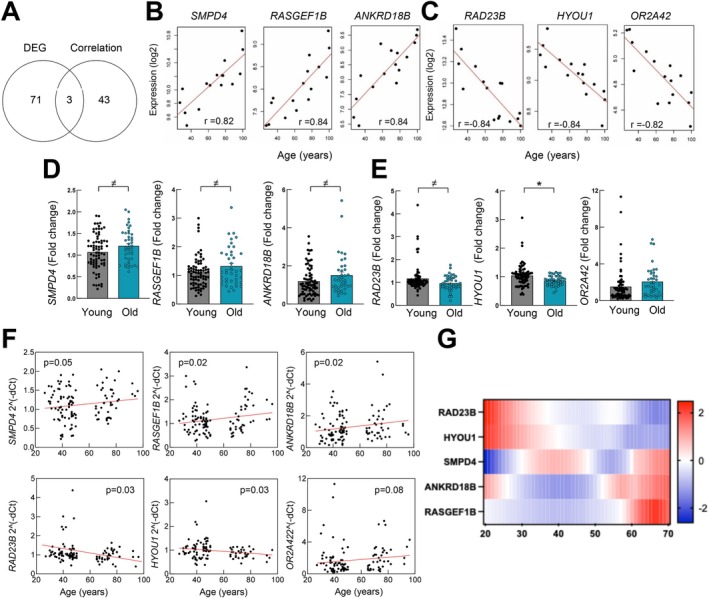
The expression of 6 genes correlates positively or negatively with chronological aging. (A) Analysis of concordant genes in the DEG and correlation study. (B, C) Correlation studies of *SMPD4*, *RASGEF1B*, *ANKRD18B*, *RAD23B*, *HYOU1*, and *OR2A42* with chronological aging in samples from the transcriptome (*n* = 16) using Pearson correlation coefficient (r) with *p* < 0.001. (D, E) mRNA expression levels of 6 genes young (*n* ≥ 75) and old individuals (*n* ≥ 39). (F) Correlation studies of *6* genes with chronological aging (*n* ≥ 114) using Pearson correlation coefficient. (^≠^ *p* < 0.1, *p < 0.05). (G) Expression of identified genes in the hippocampus in publicly available data from the Adult Genotype Tissue Expression (GTEx) project (age range 20–70).

An additional analysis was performed to identify genes whose expression correlated with chronological aging in human hippocampus. Pearson's correlation revealed that the expression of 46 genes was significantly correlated with the expression and age of the individual (*p* < 0.001), with 3 of them also identified in the DEG analysis (Figure 2A and Table S4). In this case, 6 of the genes presented a correlation coefficient higher than |0.8|. Among them, the levels of Sphingomyelin Phosphodiesterase 4 (*SMPD4*), Ras‐GEF domain‐containing family member 1B (*RASGEF1B*) and Ankyrin Repeat Domain 18B (*ANKRD18B*) correlated positively with chronological aging whereas RAD23 homolog B nucleotide excision repair protein (*RAD23B*), Hypoxia up‐regulated 1 (*HYOU1*) and Olfactory Receptor family 2 subfamily A member 42 (*OR2A42*) expression correlated negatively with age (Figure 2B,C). In order to validate these results, the mRNA expression of the 6 genes was analyzed by q‐RTPCR in additional samples from cohort 1 (120 individuals from 27 to 96 years) separated in young (*n* = 78) and old (*n* = 42) groups, finding that the expression of *SMPD4*, *RASGEF1B* and *ANKRD18B* were increased in samples from old individuals (Figure 2D), whereas the expression of *RAD23B* and *HYOU1* was significantly lower (Figure 2E). Importantly, correlation analysis confirmed that the expression of *SMPD4*, *RASGEF1B*, and *ANKRD18B* correlated positively with age (*p* = 0.04, *p* = 0.02, *p* = 0.02 respectively), while the decline in *RAD23B* and *HYOU1* levels presented a significant score (*p* = 0.02 and *p* = 0.03 respectively) (Figure 2F). In line with these results, public available data from the Adult Genotype Tissue Expression (GTEx) project showed that both RAD23B and HYOU1 expression were decreased with age within the human hippocampus (range 20–70), and SMPD4, ANKRD18B, and RASGEF1B were increased (Figure 2G). Data regarding OR2A42 was not found in the database. No marked differences were detected when the samples were divided by gender in the different genes (Figure S5). These results reveal a set of genes that correlated with chronological age.

### 3.3 The Expression of Identified Genes Is Enriched in Different Brain Regions and Cell Types

**FIGURE 3:**
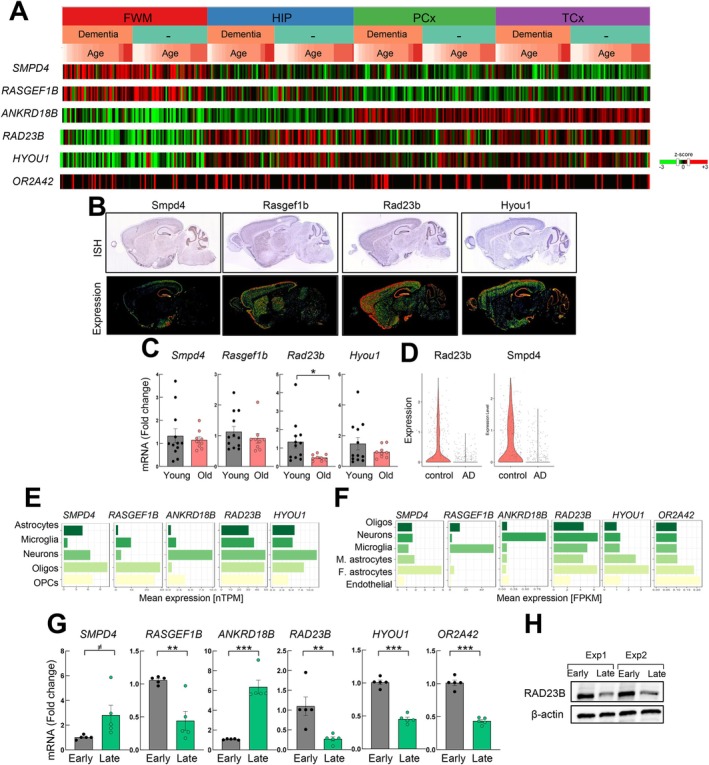
The expression of identified genes is enriched in different human and mice brain regions with age. (A) Expression of 6 genes in different brain regions from https://aging.brain‐map.org. (B) In situ hybridization (ISH, up) and expression of indicated genes from https://mouse.brain‐map.org. (C) mRNA levels of *Smpd4*, *Rasgef1b*, *Rad23b*, and *Hyou1* in the dentate gyrus (DG) of young (2 months, *n* = 12) and old (over 20 months, *n* = 9) C57BL6 mice. (D) *Rad23b* and *Smpd4* expression in AD mouse model. (E) Normalized expression (nTPM) of identified genes in indicated cell types from http://www.proteinatlas.org. (F) Expression of genes from http://www.brainrnaseq.org. (G) mRNA levels of selected genes in NHA primary astrocytes cultured at early and late passages (*n* ≥ 3). (H) RAD23 protein levels by Western Blot from 2 independent experiments at early and late stage. (^≠^ *p* < 0.1, *p < 0.05, **p < 0.01).

Taking advantage of data from public available datasets described above we characterized the expression of the six genes in different brain regions and cell types. First, data from TBI study showed that the expression of *RAD23B* and *HYOU1* appeared to be higher in the hippocampus compared to additional brain regions (Figure 3A). In contrast, the expression of *SMPD4* and *RASGEF1B* appeared to be higher in the white matter of the forebrain, whereas the levels of *ANKRD18B* were more elevated in the parietal and temporal neocortex (Figure 3A). Expression of the ortholog genes in adult C57BL6 mice brain sections from “Allen Mouse Brain Atlas”, showed that *Smpd4*, *Rasgef1b*, *Rad23b*, and *Hyou1* were detected in different regions of the brain, with the last two being highly enriched in the hippocampus (Figure 3B), paralleling the expression detected in humans. Furthermore, we studied the expression of the selected genes in hippocampus samples from a set of young and aged C57BL6 mice and we did not detected changes in the expression of *Smpd4* and *Rasgef1b* but we found significantly lower levels of *Rad23b* in old mice compared to young ones (Figure 3C). Additionally, transcriptomic public available datasets showed that some of the identified genes, mainly *Rad23b*, *Smpd4*, and *Rasgef1b*, were associated with neural stem cell populations (Figure S6A,B) (Belenguer et al. 2021; Marques‐Torrejon et al. 2021), and an additional single‐cell RNA‐Seq study revealed lower levels of *Smpd4* and *Rad23b* in mouse hippocampus with AD compared to control samples (Figure 3D). These results associate the expression of *RAD23B* to the hippocampus.

Next, we studied their expression in different brain cell types. For this, we first analyzed single‐cell RNA‐Seq from The Human Protein Atlas. We noticed that *SMPD4* and *RASGEF1* appeared to be expressed mostly by glial cells, in particular oligodendrocytes and OPCs, in contrast to *ANKRD18B* that was expressed mostly by neurons (Figure 3E). In the case of *RAD23B* and *HYOU1*, their expression pattern was similar between glial cells and neurons (Figure 3E). The additional study comparing gene expression between cell types (Zhang et al. 2016) showed that *SMPD4*, *RAD23B*, and *HYOU1* were present in the highest proportion in astrocytes, but *ANKRD18B* was mostly in neurons and *RASGEF1* in microglia (Figure 3F).

It is known that maintenance of cells for several passages under culture conditions creates a stress context that induces phenotypic and molecular changes in cells that resemble physiological aging in vivo (Tigges et al. 2014). Since the expression of the genes was mostly enriched in glial cells, we characterized the expression of the genes in human primary astrocytes (NHA cells) at early and late passages by q‐RTPCR. Paralleling the results obtained in old samples in vivo, late passage NHA presented significantly higher levels of *SMPD4* and *ANKRD18B* and lower expression of *RAD23B*, *HYOU1*, and *OR2A42* compared to early passage ones (Figure 3G). A similar decline was observed in protein RAD23B expression (Figure 3H). These results extend the link of identified genes to cellular aging.

### 3.4 RAD23B Protein Expression Diminishes With Age and With AD

**FIGURE 4:**
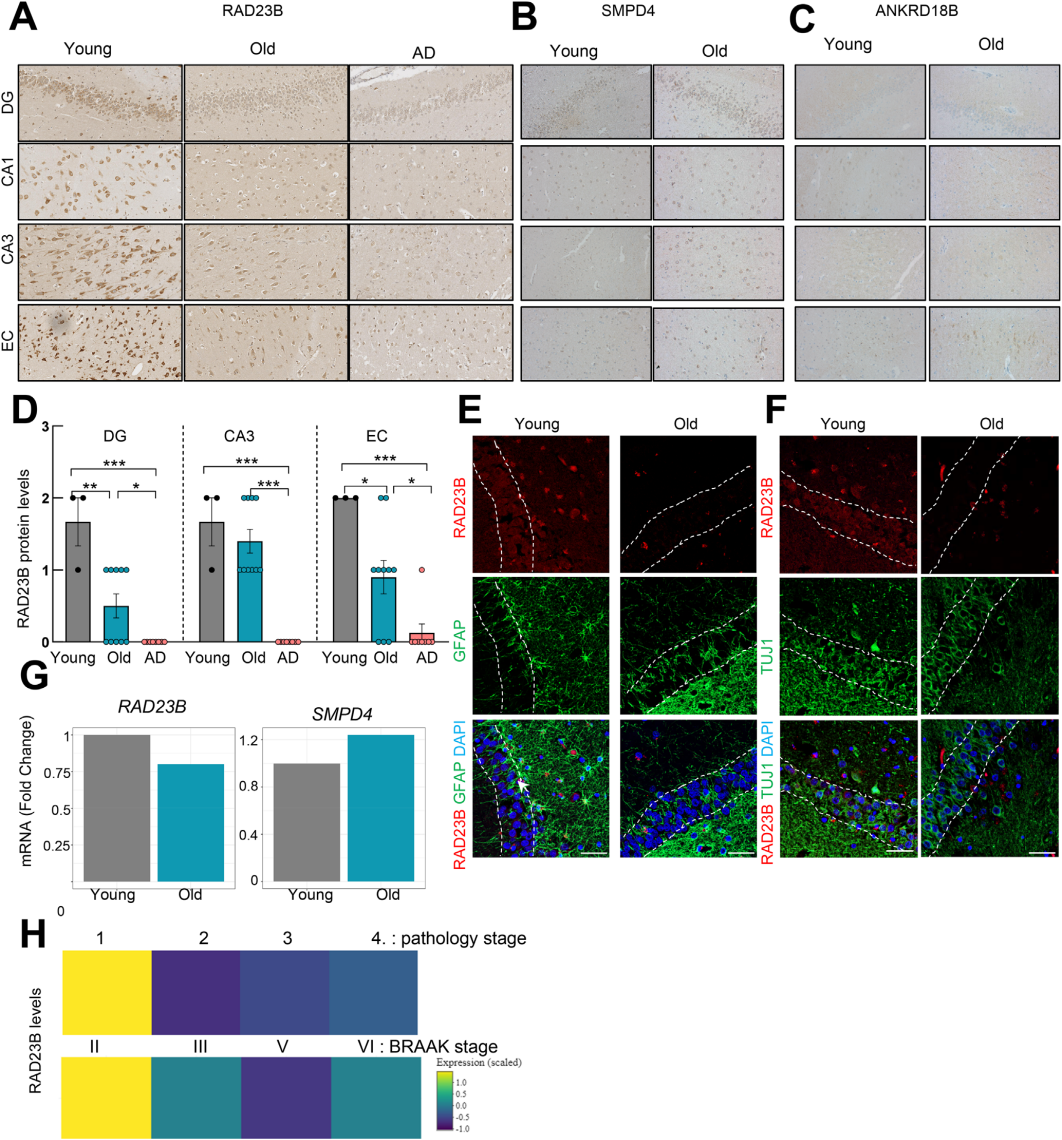
RAD23B protein expression diminishes with physiological and pathological aging. (A–D) Representative immunohistochemistry and quantification of RAD23B, SMPD4 and ANKRD18B in human dentate gyrus (DG) and cortex samples of young (*n* = 3), old (*n* = 9), and Alzheimer's disease (AD) individuals (*n* = 8). Expression was classified as high (2), moderate (1), low or absent expression (0) by pathologist. Chi‐squared test (*p < 0.05, **p < 0.01). (E–F) Representative co‐immunofluorescence of RAD23B (red) with (E) GFAP or (F) TUJ1 (green) in the DG of hippocampal coronal sections of young and old individuals (*n* = 3), (scale bar = 100 μm) arrow marks a double GFAP/RAD23b positive cell. (G) mRNA expression of *RAD23B* and *SMPD4* in human prefrontal cortical gray matter samples from old (80–94 years) versus young (24–37 years) (GSE53890). (H) Expression of *RAD23B* in astrocytes from entorhinal cortex of human samples from public available data from Alzheimer Disease Progression Atlas (https://ad‐progression‐atlas.partners.org/?page=home) taking into account BRAAK and pathology stage.

Since *RAD23B*, *SMPD4* and *ANKRD18B* were the genes that showed the strongest association with age, we focused on their characterization. Thus, we studied their protein expression in human brain samples, including the DG, CA1, and CA3 regions of the hippocampus of healthy individuals of different ages (Figure 4A–D). Notably, we detected a marked decrease in the staining of RAD23B in the different regions of the hippocampus in old samples compared to young individuals by IHC (Figure S7A), that was also detected in the cortex (Figure 4A,D). Similarly, immunofluorescence also revealed that the levels of RAD23B were lower in the hippocampus of old individuals (Figure 4E,F). On the contrary, we observed an increase in SMPD4 and ANKRD18B expression in the different regions of the hippocampus and in the cortex of old cases (Figure 4B,C). In line with these results, public available datasets from a transcriptomic study comparing human samples from old and young individuals (Lu et al. 2014), also showed higher expression of *SMPD4* and lower of *RAD23* in old cases (Figure 4G) and a similar tendency was observed in different regions of the brain, including hippocampus, in datasets from the Human Brain Transcriptome study (Figure S7C). Moreover, the expression of RAD23B in AD patients was even lower or absent in comparison with old and young healthy individuals (Figure 4A,D), and the lower levels correlated, specifically in astrocytes, with higher pathology stage and BRAAK stage on the entorhinal cortex from public available data from Alzheimer Disease Progression Atlas (Figure 4H). On the contrary, the effect was not clear with the expression of other genes (Figure S8A,B). Proportion of astrocytes expressing RAD23B, or the other genes, did not vary significantly across the stages (Figure S8C,D), suggesting that loss of *RAD23B* is due to gene downregulation rather than astrocytic loss. Altogether, these results reveal a significant decrease in the expression of RAD23B in the different areas of the brain, including the hippocampus with physiological aging that is exacerbated in pathological conditions.

To further characterize the expression of RAD23B in the different brain cell types, we completed co‐staining studies of RAD23B with glial fibrillary acidic protein (GFAP) astrocytic marker and Class III β‐Tubulin (TUJ1) neuronal marker. We observed that some of the cells positive for RAD23B were also positive for GFAP (Figure 4E) and, to a lesser extent, for TUJ1 (Figure 4F), in line with the results observed in the publicly available datasets.

### 3.5 RAD23B Silencing Impairs Astrocytic Homeostasis

**FIGURE 5:**
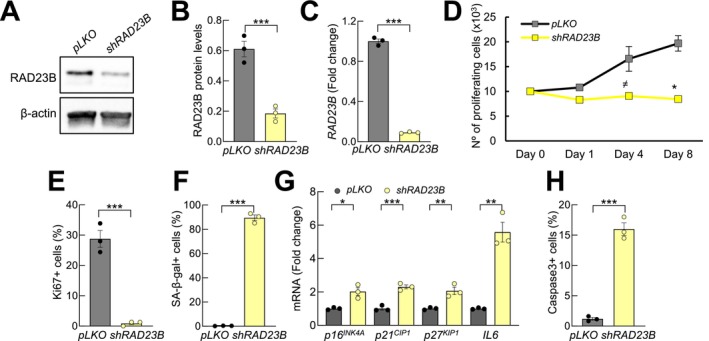
*RAD23B* silencing decreases astrocytic activity. (A, B) Representative western blot and quantification of RAD23B levels in *shRAD23B* NHA cells and controls (*pLKO*) (*n* = 3). (C) mRNA levels in same conditions (*n* = 3). (D) Cell growth in *shRAD23B* and *pLKO* astrocytes at indicated time points (*n* = 2). (E) Quantification of Ki67^+^ cells (*n* = 3). (F) SA‐β‐gal activity in *shRAD23B* and *pLKO* cells (*n* = 3). (G) mRNA expression of *p16* ^*INK4A*^, *p21* ^*CIP1*^, *p27* ^*KIP1*^, and *IL6* in *shRAD23B* and *pLKO* cells (*n* = 3). (H) Quantification of Caspase 3^+^ cells (*n* = 3). Student's *t*‐test (*p < 0.05, **p < 0.01, ***p < 0.001).

Finally, we studied the effect of *RAD23B* silencing in NHA astrocytes using lentiviral infections. We detected significantly lower protein and mRNA levels of RAD23B in astrocytes with *RAD23B* silencing compared to control ones (Figure 5A–C), thus validating our experimental model. In order to test the functional impact of *RAD23B*, we completed several experiments measuring proliferation, apoptosis, and senescence. First, we found a statistically significant reduction of cell growth measured by cell counting (Figure 5D) in *shRAD23B* cells, indicating that cell proliferation was impaired in *RAD23B*‐silenced NHA. These results were further confirmed with the lower number of positive cells for Ki67 marker (Figures 5E and S9A) as well as with the increased number of SA‐β‐gal positive cells (Figures 5F and S9B). Moreover, the expression of proliferation and senescence markers was analyzed in *shRAD23B* cells detecting a statistically significant higher expression of *p16* ^*INK4A*^, *p21* ^*CIP1*^, *p27* ^*KIP1*^, and *IL6* compared to control ones (Figure 5G). Furthermore, silencing of *RAD23B* significantly increased apoptosis measured as the number of positive cells for Caspase 3 marker (Figures 5H and S9C). These data indicate that *RAD23B* is involved in astrocyte viability and function.

## 4 Discussion

Brain aging promotes a progressive loss of mental capacities, which drives a progressive cognitive decline and functional individual deterioration that can also lead to neurodegenerative diseases (Fjell and Walhovd 2010). The identification of the molecular mechanisms underlying age‐associated cognitive decline, specifically in the hippocampus, is an area under intense investigation nowadays. For this reason, we completed a transcriptomic study in human hippocampal samples from subjects of different ages and performed 2 complementary approaches that revealed widespread molecular alterations that may underlie age‐associated cognitive decline. On the one hand, the comparison between groups of young and old individuals identified a subset of genes that were differentially expressed. Notably, they were involved in pathways related to inflammation and immune‐related pathways, DNA repair, metabolism, development, synaptic signaling, ion transport, hormone regulation, and proteostasis, all of them previously associated with aging (Lopez‐Otin et al. 2023; Ham and Lee 2020). Consistent with prior studies (Ham and Lee 2020), we observed a general upregulation of genes involved in neuroinflammation and immune‐related pathways, alongside a downregulation of genes associated with neuronal signaling and development, metabolism, and DNA repair. These findings highlight a shift in cellular states from a metabolically active during youth toward a more inflammatory and energetically constrained state in aging. On the other hand, we performed an additional bioinformatic analysis to unravel the molecular mechanisms that are associated with chronological aging and we identified changes in the expression of over 40 genes. The results of both analyses are in line with previous studies of the aging human brain that have shown dynamic gene expression changes that distinguish young adults from the aging population (Ham and Lee 2020; Wang et al. 2022).

Among the chronological aging analysis, 6 genes were the most significantly altered. *SMPD4*, *RASGEF1B*, and *ANKRD18B* correlated positively with age whilst the expression of *RAD23B*, *HYOU1*, and *OR2A42* showed a decline with age. These genes are involved in metabolism, DNA repair and protein regulation further highlighting the association of these biological processes with brain aging. *SMPD4* has a role in the homeostasis of membrane sphingolipids, and influences membrane integrity, and endoplasmic reticulum (ER) organization and function (Krut et al. 2006). In skeletal muscle, it mediates TNF‐stimulated oxidant production and diseases associated with *SMPD4* include neurodevelopmental disorder (Corcoran et al. 2008). *RASGEF1B*, a toll‐like receptor‐inducible Ras guanine‐nucleotide exchange factor, has been associated with cancer, proliferation, and inflammation pathways (Andrade et al. 2010), whereas *ANKRD18B* participates in cell growth, cell cycle regulation, and signal transduction and it has been linked with cancer progression (Liu et al. 2015). *HYOU1* plays an important role in protein folding and secretion in the ER (Rao et al. 2021). Its expression is upregulated in many diseases, including different types of cancer and ER stress‐related diseases. These genes have not been linked directly to the aging process and little is known regarding their activity in the brain; however, our data and their described functions suggest that they could be involved in different biological processes underlying brain aging.

Emerging single‐cell RNA sequencing and spatial transcriptomic studies have demonstrated that transcriptomic aging signatures vary substantially between hippocampal subregions (CA1, CA3, dentate gyrus) and cell types (Ham and Lee 2020; Wu et al. 2025; Su et al. 2023; Zhou et al. 2022). Our bulk tissue results likely reflect a composite of these heterogeneous changes. Indeed, we identified genes differentially and specifically expressed in different progenitor, glial, and neuronal cell types and they appeared to be conserved across the diverse hippocampal compartments. Moreover, data from several publicly available studies indicate that the observed differences in expression are not confined to the hippocampus region but are also extended across additional brain regions.

Among the candidate genes, we focused on *RAD23B*, since it showed the strongest differences at statistical level both in human and mice. *RAD23B* gene is involved in DNA damage repair, specifically in nucleotide excision repair (NER), a multistep process that corrects DNA alterations from endogenous oxidative stress or single‐strand breaks, among others (Katiyar and Lennarz 2005). In addition to his role in DNA damage recognition, *RAD23B* also has an important function in protein degradation (it binds ubiquitinated substrates and the proteasome) and in cell cycle control, which are also relevant processes in aging (Chen and Madura 2006). Notably, IHC, IF and public datasets revealed that RAD23B protein levels were lower in different regions of the hippocampus and the brain of aged individuals with physiological aging. In addition, we identified that RAD23B protein expression was even lower or totally absent in patients with AD in comparison with old or young samples, and expression was reduced in astrocytes of entorhinal cortex with progression of the pathology, indicating that it could be a negative marker of physiological or pathological aging. Consistent with this, RAD23B protein inclusions have been found in several brain regions in a number of neurodegenerative diseases, including frontotemporal dementia, Huntington's disease, spinocerebellar ataxia type 3 and 7, fragile X associated tremor/ataxia syndrome and Parkinson's disease (Riemslagh et al. 2019; Bergink et al. 2006). The in vitro results suggest that *RAD23B* is involved in astrocyte viability and activity, data reinforced by the expression studies in samples with physiological aging and specially with AD. Our results further reinforce the link between DNA repair pathways and brain aging. In this sense, DNA damage gives rise to genomic instability and induces signaling cascades leading to cell death, senescence or secretion of inflammatory cytokines (Rodier et al. 2009; Wong and Chow 2023). Moreover, mutations in DNA repair genes such as *BRCA1*, *ATM* or *RAD51* have been reported in AD (Lin et al. 2020), as well as accumulation of the marker of DNA damage gamma‐H2A histone X in astrocytes of hippocampus and cerebral cortex of patients (Myung et al. 2008).

While our data provide a comprehensive overview of hippocampal transcriptional aging, several limitations should be acknowledged including the limited number of samples where the transcriptome study was completed or the bulk analysis that lacks the resolution to distinguish cell‐type–specific responses. Additionally, the sample obtention for the transcriptome study from forensic autopsies had limitations such as no information of the specific hippocampal subregions analyzed. Moreover, transcriptomic alterations may not directly correspond to changes at the functional level in cognition, information that was not available. In summary, our study performed 2 complementary approaches, which identified transcriptome dynamics with aging with differentially expressed genes and biological processes in human hippocampus. Our findings reinforce the view that aging profoundly reshapes the hippocampal transcriptome, shifting the molecular landscape toward inflammation and reduced resilience. Among the molecular alterations, we described the decline in the expression of RAD23B with aging that is exacerbated in AD pathology becoming a potential biomarker and driver of physiological and pathological aging in the brain.

## Author Contributions

A.S.‐A. performed most of the molecular experiments, analyzed the results and wrote the draft of the manuscript. M.M.‐V. completed the stainings in human samples with help of J.L. J.V.‐A. helped with the in vitro studies. S.C.‐S. and J.L. completed the studies with public available datasets. M.A.‐I. and M.M.‐V. provided biological samples and M.A.‐I., M.M.‐V., and J.L. recopilated clinical information. M.M.‐C., A.A., and D.O. performed the transcriptomic analysis. A.M. directed and coordinated the project, obtained funding and wrote the manuscript.

## Funding

This work was supported by Instituto de Salud Carlos III (ISCIII) through the project (PI19/01355, PI22/01905, FORT23‐00026, DTS24/00153, PI25/01917) and co‐funded by the European Union, Diputacion Foral Gipuzkoa—Adinberri (FADIN19/001, FA547/2022) and Health Department of the Basque Country (2022111069).

## Conflicts of Interest

The authors declare no conflicts of interest.

## Supporting information


**Figure S1:** (A, B) mRNA expression levels of 33 genes in young (*n* = 15) and old individuals (*n* = 16). Statistical significance is determined as: (≠ *p* < 0.1, * *p* < 0.05, ** *p* < 0.01, *** *p* < 0.001).
**Figure S2:** Sex‐specific mRNA expression levels of 33 target genes. Data are shown for males (A, C; young, *n* = 8; old, *n* = 9) and females (B, D; young, *n* = 7; old, *n* = 7). Statistical significance is determined as: (≠ *p* < 0.1, * *p* < 0.05).
**Figure S3:** Spatial expression patterns of orthologous genes in the adult mouse brain. (A, B) Representative in situ hybridization (ISH) images and expression maps from Allen Mouse Brain Atlas demonstrating the enrichment of candidate genes within the dentate gyrus (DG) of adult C57BL/6 mice.
**Figure S4:**. Cell‐type specific expression of candidate genes in the human brain. (A, B) Single‐cell RNA‐sequencing (scRNA‐seq) analysis from The Human Protein Atlas and Zhang et al. (2016).
**Figure S5:** Sex‐specific age related mRNA expression and correlation analysis of 6 selected genes. (A, B) mRNA expression levels of six candidate genes in males (young, *n* = 63; old, *n* = 27) and females (young, *n* = 12, old, *n* = 12). (C, D) Correlation studies of 6 genes with chronological aging in males and females separately. Statistical significance is determined as: (≠ *p* < 0.1, * *p* < 0.05).
**Figure S6:** Association of candidate genes with neural stem cell (NSC) populations. Heatmaps representing mRNA expression levels at cell level of candidate genes across NSC and progenitor clusters. Data obtained from gene expression omnibus (GEO) with accession number GSE138243 (A) and GSE168189 (B).
**Figure S7:** (A) Low magnification of RAD23b staining for young, old and neurodegenerative hippocampal brain slices. CA = cornus ammonis, DG = dentate gyrus. (B) Expression of RAD23B and SMPD4 in different brain regions across age. Data extracted from Human Brain Transcriptome. AMY = amygdala, CBC = cerebellar cortex, HIP = hippocampus, MD = mediodorsal nucleus of the thalamus, NCX = neocortex, STR = striatum.
**Figure S8:** Expression profile of candidate genes with Alzheimer disease progression. (A, B) Expression levels of the six candidate genes in astrocytes of entorhinal cortex categorized by (A) pathology stage and (B) Braak stage. (C, D) Bubble charts illustrating the proportion of astrocytes expressing RAD23B and the other candidate genes across (C) pathological stages and (D) Braak stages. Proportion of astrocytes (GFAP positive) expressing RAD23b is written under the bubble chart. Data obtained from Alzheimer Disease Progression Atlas.
**Figure S9:** Silencing of RAD23B in astrocytes triggers cellular senescence and apoptosis. Representative images for (A) Ki67, (B) SA‐β‐galactosidase and (C) Caspase 3 for either pLKO or shRAD23B infected cells.

**Table S1:** Information of the human hippocampus samples used for the transcriptomic study (cohort 1) divided by groups.
**Table S2:** Information of the human hippocampus samples used for mRNA validations (extension of cohort 1) divided by groups.
**Table S3:** Information of the human hippocampus samples (cohort 2) used for protein studies (immunofluorescence and immunohistochemistry) divided by groups.

**Table S4:** acel70459‐sup‐0003‐Table4.xlsx.

## Data Availability

The data that support the findings of this study are openly available in NCBI's Gene Expression Omnibus at https://www.ncbi.nlm.nih.gov/geo/, reference number GSE201118.

## References

[acel70459-bib-0001] Aimone, J. B. , Y. Li , S. W. Lee , G. D. Clemenson , W. Deng , and F. H. Gage . 2014. “Regulation and Function of Adult Neurogenesis: From Genes to Cognition.” Physiological Reviews 94, no. 4: 991–1026. 25287858 10.1152/physrev.00004.2014 PMC4280160

[acel70459-bib-0002] Andrade, W. A. , A. M. Silva , V. S. Alves , et al. 2010. “Early Endosome Localization and Activity of RasGEF1b, a Toll‐Like Receptor‐Inducible Ras Guanine‐Nucleotide Exchange Factor.” Genes and Immunity 11, no. 6: 447–457. 20090772 10.1038/gene.2009.107

[acel70459-bib-0003] Arrizabalaga, O. , L. Moreno‐Cugnon , J. Auzmendi‐Iriarte , et al. 2017. “High Expression of MKP1/DUSP1 Counteracts Glioma Stem Cell Activity and Mediates HDAC Inhibitor Response.” Oncogene 6, no. 12: 401. 10.1038/s41389-017-0003-9 PMC5865544 29284798

[acel70459-bib-0004] Belenguer, G. , P. Duart‐Abadia , A. Jordan‐Pla , et al. 2021. “Adult Neural Stem Cells Are Alerted by Systemic Inflammation Through TNF‐Alpha Receptor Signaling.” Cell Stem Cell 28, no. 2: 285–299.e9. 33207218 10.1016/j.stem.2020.10.016

[acel70459-bib-0005] Bergink, S. , L. A. Severijnen , N. Wijgers , et al. 2006. “The DNA Repair‐Ubiquitin‐Associated HR23 Proteins Are Constituents of Neuronal Inclusions in Specific Neurodegenerative Disorders Without Hampering DNA Repair.” Neurobiology of Disease 23, no. 3: 708–716. 16860562 10.1016/j.nbd.2006.06.005

[acel70459-bib-0006] Bishop, N. A. , T. Lu , and B. A. Yankner . 2010. “Neural Mechanisms of Ageing and Cognitive Decline.” Nature 464, no. 7288: 529–535. 20336135 10.1038/nature08983 PMC2927852

[acel70459-bib-0007] Carrasco‐Garcia, E. , L. Moreno‐Cugnon , I. Garcia , et al. 2019. “SOX2 Expression Diminishes With Ageing in Several Tissues in Mice and Humans.” Mechanisms of Ageing and Development 177: 30–36. 29574045 10.1016/j.mad.2018.03.008

[acel70459-bib-0008] Chen, L. , and K. Madura . 2006. “Evidence for Distinct Functions for Human DNA Repair Factors hHR23A and hHR23B.” FEBS Letters 580, no. 14: 3401–3408. 16712842 10.1016/j.febslet.2006.05.012

[acel70459-bib-0009] Consortium GT . 2020. “The GTEx Consortium Atlas of Genetic Regulatory Effects Across Human Tissues.” Science 369, no. 6509: 1318–1330. 32913098 10.1126/science.aaz1776 PMC7737656

[acel70459-bib-0010] Corcoran, C. A. , Q. He , S. Ponnusamy , B. Ogretmen , Y. Huang , and M. S. Sheikh . 2008. “Neutral Sphingomyelinase‐3 Is a DNA Damage and Nongenotoxic Stress‐Regulated Gene That Is Deregulated in Human Malignancies.” Molecular Cancer Research 6, no. 5: 795–807. 18505924 10.1158/1541-7786.MCR-07-2097 PMC2642592

[acel70459-bib-0011] Fjell, A. M. , and K. B. Walhovd . 2010. “Structural Brain Changes in Aging: Courses, Causes and Cognitive Consequences.” Reviews in the Neurosciences 21, no. 3: 187–221. 20879692 10.1515/revneuro.2010.21.3.187

[acel70459-bib-0012] Ham, S. , and S. V. Lee . 2020. “Advances in Transcriptome Analysis of Human Brain Aging.” Experimental and Molecular Medicine 52, no. 11: 1787–1797. 33244150 10.1038/s12276-020-00522-6 PMC8080664

[acel70459-bib-0013] Higgins‐Chen, A. T. , K. L. Thrush , and M. E. Levine . 2021. “Aging Biomarkers and the Brain.” Seminars in Cell & Developmental Biology 116: 180–193. 33509689 10.1016/j.semcdb.2021.01.003 PMC8292153

[acel70459-bib-0014] Isaev, N. K. , E. E. Genrikhs , M. V. Oborina , and E. V. Stelmashook . 2018. “Accelerated Aging and Aging Process in the Brain.” Reviews in the Neurosciences 29, no. 3: 233–240. 29150992 10.1515/revneuro-2017-0051

[acel70459-bib-0015] Jiang, J. , C. Wang , R. Qi , H. Fu , and Q. Ma . 2020. “scREAD: A Single‐Cell RNA‐Seq Database for Alzheimer's Disease.” iScience 23, no. 11: 101769. 33241205 10.1016/j.isci.2020.101769 PMC7674513

[acel70459-bib-0016] Katiyar, S. , and W. J. Lennarz . 2005. “Studies on the Intracellular Localization of hHR23B.” Biochemical and Biophysical Research Communications 337, no. 4: 1296–1300. 16253613 10.1016/j.bbrc.2005.09.192

[acel70459-bib-0017] Krut, O. , K. Wiegmann , H. Kashkar , B. Yazdanpanah , and M. Krönke . 2006. “Novel Tumor Necrosis Factor‐Responsive Mammalian Neutral Sphingomyelinase‐3 Is a C‐Tail‐Anchored Protein.” Journal of Biological Chemistry 281, no. 19: 13784–13793. 16517606 10.1074/jbc.M511306200

[acel70459-bib-0018] Lin, X. , A. Kapoor , Y. Gu , et al. 2020. “Contributions of DNA Damage to Alzheimer's Disease.” International Journal of Molecular Sciences 21, no. 5: 1666. 32121304 10.3390/ijms21051666 PMC7084447

[acel70459-bib-0019] Lisman, J. , G. Buzsaki , H. Eichenbaum , L. Nadel , C. Ranganath , and A. D. Redish . 2017. “Viewpoints: How the Hippocampus Contributes to Memory, Navigation and Cognition.” Nature Neuroscience 20, no. 11: 1434–1447. 29073641 10.1038/nn.4661 PMC5943637

[acel70459-bib-0020] Liu, W. B. , F. Han , X. Jiang , et al. 2015. “Epigenetic Regulation of ANKRD18B in Lung Cancer.” Molecular Carcinogenesis 54, no. 4: 312–321. 24249358 10.1002/mc.22101

[acel70459-bib-0021] Lopez‐Otin, C. , M. A. Blasco , L. Partridge , M. Serrano , and G. Kroemer . 2023. “Hallmarks of Aging: An Expanding Universe.” Cell 186, no. 2: 243–278. 36599349 10.1016/j.cell.2022.11.001

[acel70459-bib-0022] Lu, T. , L. Aron , J. Zullo , et al. 2014. “REST and Stress Resistance in Ageing and Alzheimer's Disease.” Nature 507, no. 7493: 448–454. 24670762 10.1038/nature13163 PMC4110979

[acel70459-bib-0023] Lu, T. , Y. Pan , S. Y. Kao , et al. 2004. “Gene Regulation and DNA Damage in the Ageing Human Brain.” Nature 429, no. 6994: 883–891. 15190254 10.1038/nature02661

[acel70459-bib-0024] Marques‐Torrejon, M. A. , C. A. C. Williams , B. Southgate , et al. 2021. “LRIG1 Is a Gatekeeper to Exit From Quiescence in Adult Neural Stem Cells.” Nature Communications 12, no. 1: 2594. 10.1038/s41467-021-22813-w PMC8110534 33972529

[acel70459-bib-0025] Matheu, A. , P. Klatt , and M. Serrano . 2005. “Regulation of the INK4a/ARF Locus by Histone Deacetylase Inhibitors.” Journal of Biological Chemistry 280, no. 51: 42433–42441. 16251190 10.1074/jbc.M508270200

[acel70459-bib-0026] Mattson, M. P. , and T. V. Arumugam . 2018. “Hallmarks of Brain Aging: Adaptive and Pathological Modification by Metabolic States.” Cell Metabolism 27, no. 6: 1176–1199. 29874566 10.1016/j.cmet.2018.05.011 PMC6039826

[acel70459-bib-0027] Moreno‐Valladares, M. , L. Moreno‐Cugnon , T. M. Silva , et al. 2020. “CD8(+) T Cells Are Increased in the Subventricular Zone With Physiological and Pathological Aging.” Aging Cell 19, no. 9: e13198. 32741087 10.1111/acel.13198 PMC7511866

[acel70459-bib-0028] Murman, D. L. 2015. “The Impact of Age on Cognition.” Seminars in Hearing 36, no. 3: 111–121. 27516712 10.1055/s-0035-1555115 PMC4906299

[acel70459-bib-0029] Myung, N. H. , X. Zhu , I. I. Kruman , et al. 2008. “Evidence of DNA Damage in Alzheimer Disease: Phosphorylation of Histone H2AX in Astrocytes.” Age (Dordrecht, Netherlands) 30, no. 4: 209–215. 19424844 10.1007/s11357-008-9050-7 PMC2585649

[acel70459-bib-0030] Navarro Negredo, P. , R. W. Yeo , and A. Brunet . 2020. “Aging and Rejuvenation of Neural Stem Cells and Their Niches.” Cell Stem Cell 27, no. 2: 202–223. 32726579 10.1016/j.stem.2020.07.002 PMC7415725

[acel70459-bib-0031] Rao, S. , L. Oyang , J. Liang , et al. 2021. “Biological Function of HYOU1 in Tumors and Other Diseases.” Oncotargets and Therapy 14: 1727–1735. 33707955 10.2147/OTT.S297332 PMC7943547

[acel70459-bib-0032] Riemslagh, F. W. , H. Lans , H. Seelaar , et al. 2019. “HR23B Pathology Preferentially Co‐Localizes With p62, pTDP‐43 and Poly‐GA in C9ORF72‐Linked Frontotemporal Dementia and Amyotrophic Lateral Sclerosis.” Acta Neuropathologica Communications 7, no. 1: 39. 30867060 10.1186/s40478-019-0694-6 PMC6416930

[acel70459-bib-0033] Rodier, F. , J. P. Coppé , C. K. Patil , et al. 2009. “Persistent DNA Damage Signalling Triggers Senescence‐Associated Inflammatory Cytokine Secretion.” Nature Cell Biology 11, no. 8: 973–979. 19597488 10.1038/ncb1909 PMC2743561

[acel70459-bib-0034] Saenz‐Antonanzas, A. , M. Munoz‐Culla , P. Rigo , et al. 2024. “Centenarian Hippocampus Displays High Levels of Astrocytic Metallothioneins.” Aging Cell 23: e14201. 38769809 10.1111/acel.14201 PMC11320342

[acel70459-bib-0035] Serrano‐Pozo, A. , H. Li , Z. Li , et al. 2024. “Astrocyte Transcriptomic Changes Along the Spatiotemporal Progression of Alzheimer's Disease.” Nature Neuroscience 27, no. 12: 2384–2400. 39528672 10.1038/s41593-024-01791-4 PMC11614739

[acel70459-bib-0036] Small, S. A. , S. A. Schobel , R. B. Buxton , M. P. Witter , and C. A. Barnes . 2011. “A Pathophysiological Framework of Hippocampal Dysfunction in Ageing and Disease.” Nature Reviews. Neuroscience 12, no. 10: 585–601. 21897434 10.1038/nrn3085 PMC3312472

[acel70459-bib-0037] Soreq, L. , J. Rose , E. Soreq , et al. 2017. “Major Shifts in Glial Regional Identity Are a Transcriptional Hallmark of Human Brain Aging.” Cell Reports 18, no. 2: 557–570. 28076797 10.1016/j.celrep.2016.12.011 PMC5263238

[acel70459-bib-0038] Su, Y. , Y. Zhou , M. L. Bennett , et al. 2023. “A Single‐Cell Transcriptome Atlas of Glial Diversity in the Human Hippocampus Across the Postnatal Lifespan.” Cell Stem Cell 30, no. 1: 113. 36608676 10.1016/j.stem.2022.12.007 PMC9901278

[acel70459-bib-0039] Tigges, J. , J. Krutmann , E. Fritsche , et al. 2014. “The Hallmarks of Fibroblast Ageing.” Mechanisms of Ageing and Development 138: 26–44. 24686308 10.1016/j.mad.2014.03.004

[acel70459-bib-0040] Wang, W. , M. Wang , M. Yang , et al. 2022. “Transcriptome Dynamics of Hippocampal Neurogenesis in Macaques Across the Lifespan and Aged Humans.” Cell Research 32, no. 8: 729–743. 35750757 10.1038/s41422-022-00678-y PMC9343414

[acel70459-bib-0041] Wong, G. C. , and K. H. Chow . 2023. “DNA Damage Response‐Associated Cell Cycle Re‐Entry and Neuronal Senescence in Brain Aging and Alzheimer's Disease.” Journal of Alzheimer's Disease 94, no. s1: S429–S451. 10.3233/JAD-220203 PMC10473156 35848025

[acel70459-bib-0042] Wu, Y. , V. I. Korobeynyk , M. Zamboni , et al. 2025. “Multimodal Transcriptomics Reveal Neurogenic Aging Trajectories and Age‐Related Regional Inflammation in the Dentate Gyrus.” Nature Neuroscience 28, no. 2: 415–430. 39762661 10.1038/s41593-024-01848-4 PMC11802457

[acel70459-bib-0043] Zhang, Y. , S. A. Sloan , L. E. Clarke , et al. 2016. “Purification and Characterization of Progenitor and Mature Human Astrocytes Reveals Transcriptional and Functional Differences With Mouse.” Neuron 89, no. 1: 37–53. 26687838 10.1016/j.neuron.2015.11.013 PMC4707064

[acel70459-bib-0044] Zhong, S. , M. Wang , Y. Zhan , et al. 2020. “Single‐Nucleus RNA Sequencing Reveals Transcriptional Changes of Hippocampal Neurons in APP23 Mouse Model of Alzheimer's Disease.” Bioscience, Biotechnology, and Biochemistry 84, no. 5: 919–926. 31928331 10.1080/09168451.2020.1714420

[acel70459-bib-0045] Zhou, Y. , Y. Su , S. Li , et al. 2022. “Molecular Landscapes of Human Hippocampal Immature Neurons Across Lifespan.” Nature 607, no. 7919: 527–533. 35794479 10.1038/s41586-022-04912-w PMC9316413

