# Supplementary material for: Differential Gene Expression in Human Hippocampus With Aging
Source: Aging Cell. 2026 Mar 26;25(4):e70459. doi: 10.1111/acel.70459 (PMC13140639; doi:10.1111/acel.70459)
Supplement: Supplementary file 1 — Figure S1: (A, B) mRNA expression levels of 33 genes in young (n = 15) and old individuals (n = 16). Statistical significance is determined as: (≠ p < 0.1, * p < 0.05, ** p < 0.01, *** p < 0.001). Figure S2: Sex‐specific mRNA expression levels of 33 target genes. Data are shown for males (A, C; young, n = 8; old, n = 9) and females (B, D; young, n = 7; old, n = 7). Statistical significance is determined as: (≠ p < 0.1, * p < 0.05). Figure S3: Spatial expression patterns of orthologous genes in the adult mouse brain. (A, B) Representative in situ hybridization (ISH) images and expression maps from Allen Mouse Brain Atlas demonstrating the enrichment of candidate genes within the dentate gyrus (DG) of adult C57BL/6 mice. Figure S4:. Cell‐type specific expression of candidate genes in the human brain. (A, B) Single‐cell RNA‐sequencing (scRNA‐seq) analysis from The Human Protein Atlas and Zhang et al. (2016). Figure S5: Sex‐specific age related mRNA expression and correlation analysis of 6 selected genes. (A, B) mRNA expression levels of six candidate genes in males (young, n = 63; old, n = 27) and females (young, n = 12, old, n = 12). (C, D) Correlation studies of 6 genes with chronological aging in males and females separately. Statistical significance is determined as: (≠ p < 0.1, * p < 0.05). Figure S6: Association of candidate genes with neural stem cell (NSC) populations. Heatmaps representing mRNA expression levels at cell level of candidate genes across NSC and progenitor clusters. Data obtained from gene expression omnibus (GEO) with accession number GSE138243 (A) and GSE168189 (B). Figure S7: (A) Low magnification of RAD23b staining for young, old and neurodegenerative hippocampal brain slices. CA = cornus ammonis, DG = dentate gyrus. (B) Expression of RAD23B and SMPD4 in different brain regions across age. Data extracted from Human Brain Transcriptome. AMY = amygdala, CBC = cerebellar cortex, HIP = hippocampus, MD = mediodorsal nucleus of the thalamus, NCX = [file ACEL-25-e70459-s001.pptx]

## Slide 1
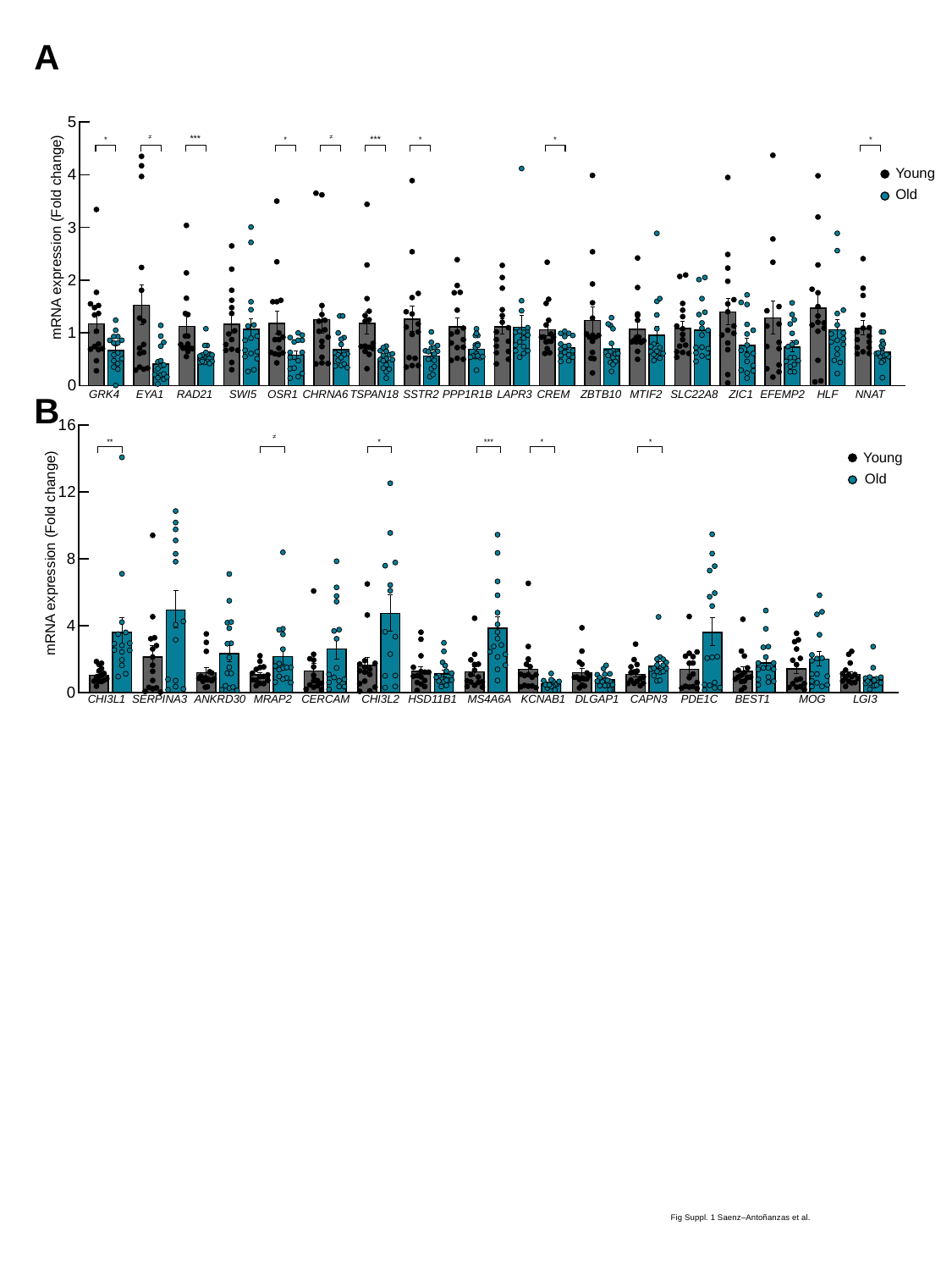

A
***
***
≠
≠
*
*
*
*
*
Young
Old
mRNA expression (Fold change)
GRK4
EYA1
RAD21
SWI5
OSR1
CHRNA6
TSPAN18
SSTR2
PPP1R1B
LAPR3
CREM
ZBTB10
MTIF2
SLC22A8
ZIC1
EFEMP2
HLF
NNAT
B
≠
**
*
***
*
*
Young
Old
mRNA expression (Fold change)
CHI3L1
SERPINA3
ANKRD30
MRAP2
CHI3L2
CERCAM
HSD11B1
MS4A6A
KCNAB1
DLGAP1
CAPN3
PDE1C
BEST1
MOG
LGI3
Fig Suppl. 1 Saenz–Antoñanzas et al.

## Slide 2
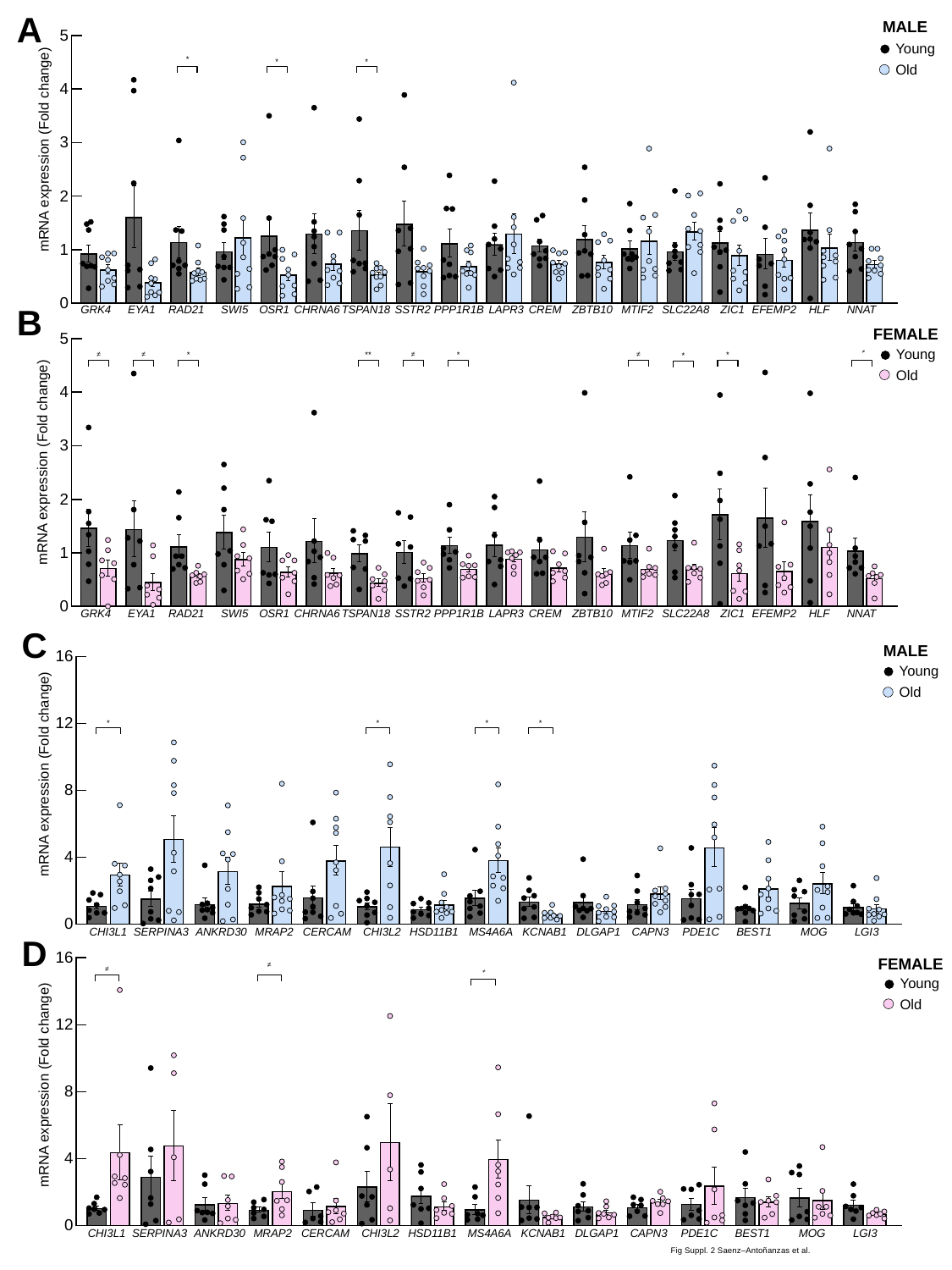

A
MALE
Young
Old
*
*
*
mRNA expression (Fold change)
B
GRK4
EYA1
RAD21
SWI5
OSR1
CHRNA6
TSPAN18
SSTR2
PPP1R1B
LAPR3
CREM
ZBTB10
MTIF2
SLC22A8
ZIC1
EFEMP2
HLF
NNAT
FEMALE
Young
Old
≠
≠
≠
*
**
≠
*
≠
*
*
mRNA expression (Fold change)
GRK4
EYA1
RAD21
SWI5
OSR1
CHRNA6
TSPAN18
SSTR2
PPP1R1B
LAPR3
CREM
ZBTB10
MTIF2
SLC22A8
ZIC1
EFEMP2
HLF
NNAT
C
MALE
Young
Old
*
*
*
*
mRNA expression (Fold change)
CHI3L1
SERPINA3
ANKRD30
MRAP2
CHI3L2
CERCAM
HSD11B1
MS4A6A
KCNAB1
DLGAP1
CAPN3
PDE1C
BEST1
MOG
LGI3
D
FEMALE
≠
≠
≠
Young
Old
mRNA expression (Fold change)
CHI3L1
SERPINA3
ANKRD30
MRAP2
CHI3L2
CERCAM
HSD11B1
MS4A6A
KCNAB1
DLGAP1
CAPN3
PDE1C
BEST1
MOG
LGI3
Fig Suppl. 2 Saenz–Antoñanzas et al.

## Slide 3
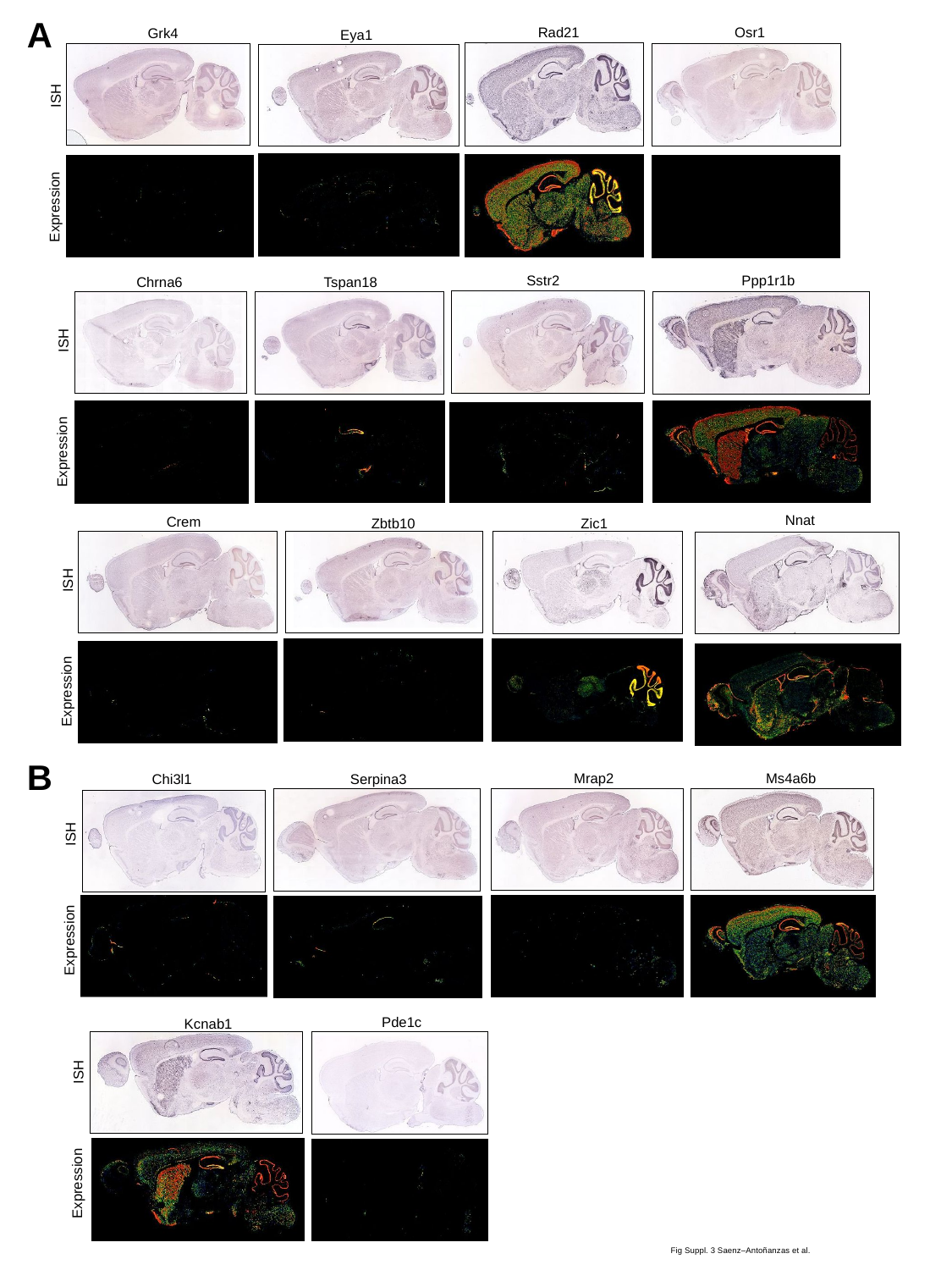

A
Osr1
Rad21
Grk4
Eya1
ISH
Expression
Ppp1r1b
Sstr2
Tspan18
Chrna6
ISH
Expression
Nnat
Crem
Zbtb10
Zic1
ISH
Expression
B
Mrap2
Ms4a6b
Chi3l1
Serpina3
ISH
Expression
Chi3l1
Pde1c
Kcnab1
ISH
Expression
Fig Suppl. 3 Saenz–Antoñanzas et al.

## Slide 4
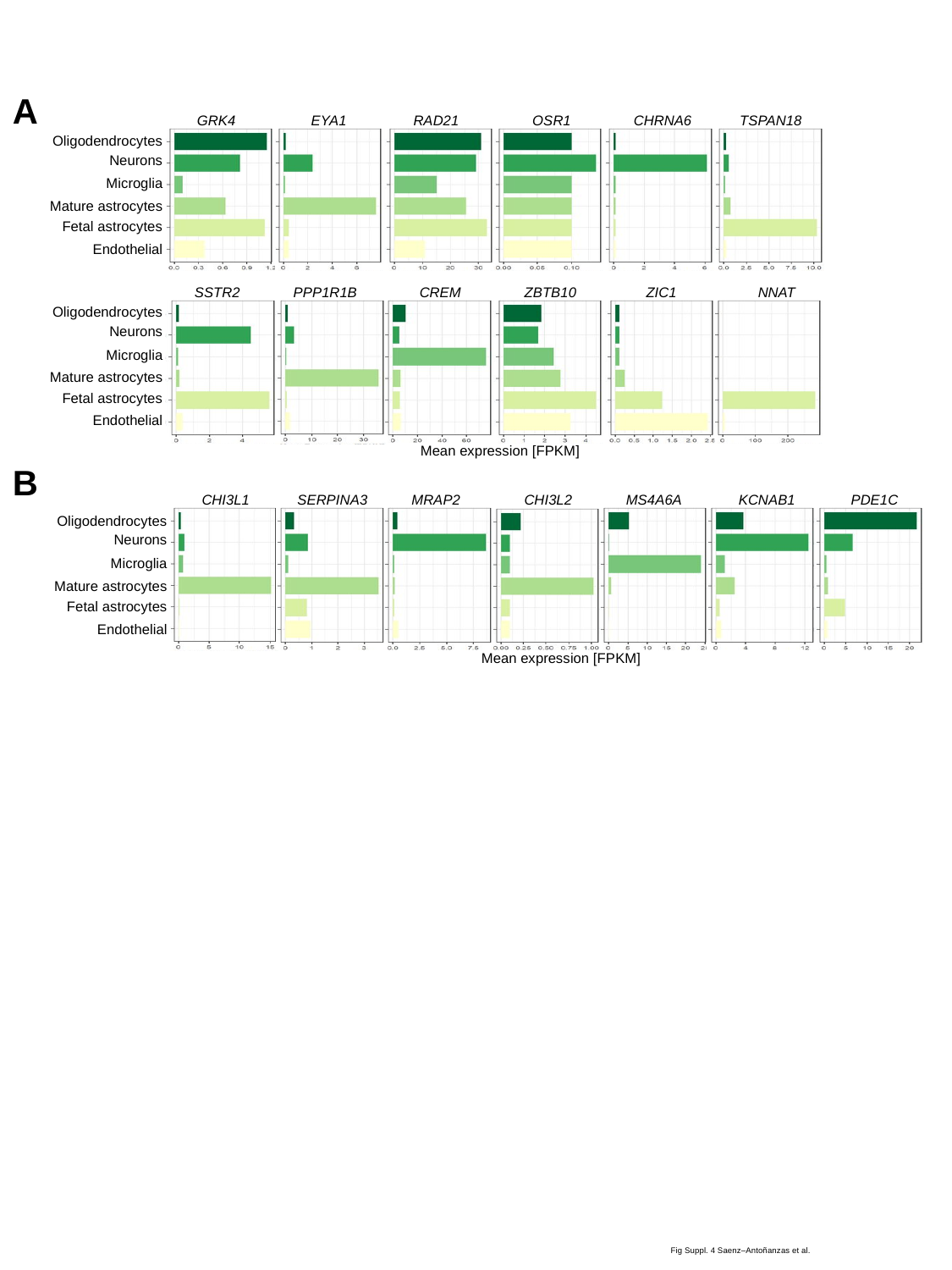

A
GRK4
EYA1
RAD21
OSR1
CHRNA6
TSPAN18
Oligodendrocytes
Neurons
Microglia
Mature astrocytes
Fetal astrocytes
Endothelial
SSTR2
PPP1R1B
CREM
ZBTB10
ZIC1
NNAT
Oligodendrocytes
Neurons
Microglia
Mature astrocytes
Fetal astrocytes
Endothelial
Mean expression [FPKM]
B
CHI3L1
SERPINA3
MRAP2
CHI3L2
MS4A6A
KCNAB1
PDE1C
Oligodendrocytes
Neurons
Microglia
Mature astrocytes
Fetal astrocytes
Endothelial
Mean expression [FPKM]
Fig Suppl. 4 Saenz–Antoñanzas et al.

## Slide 5
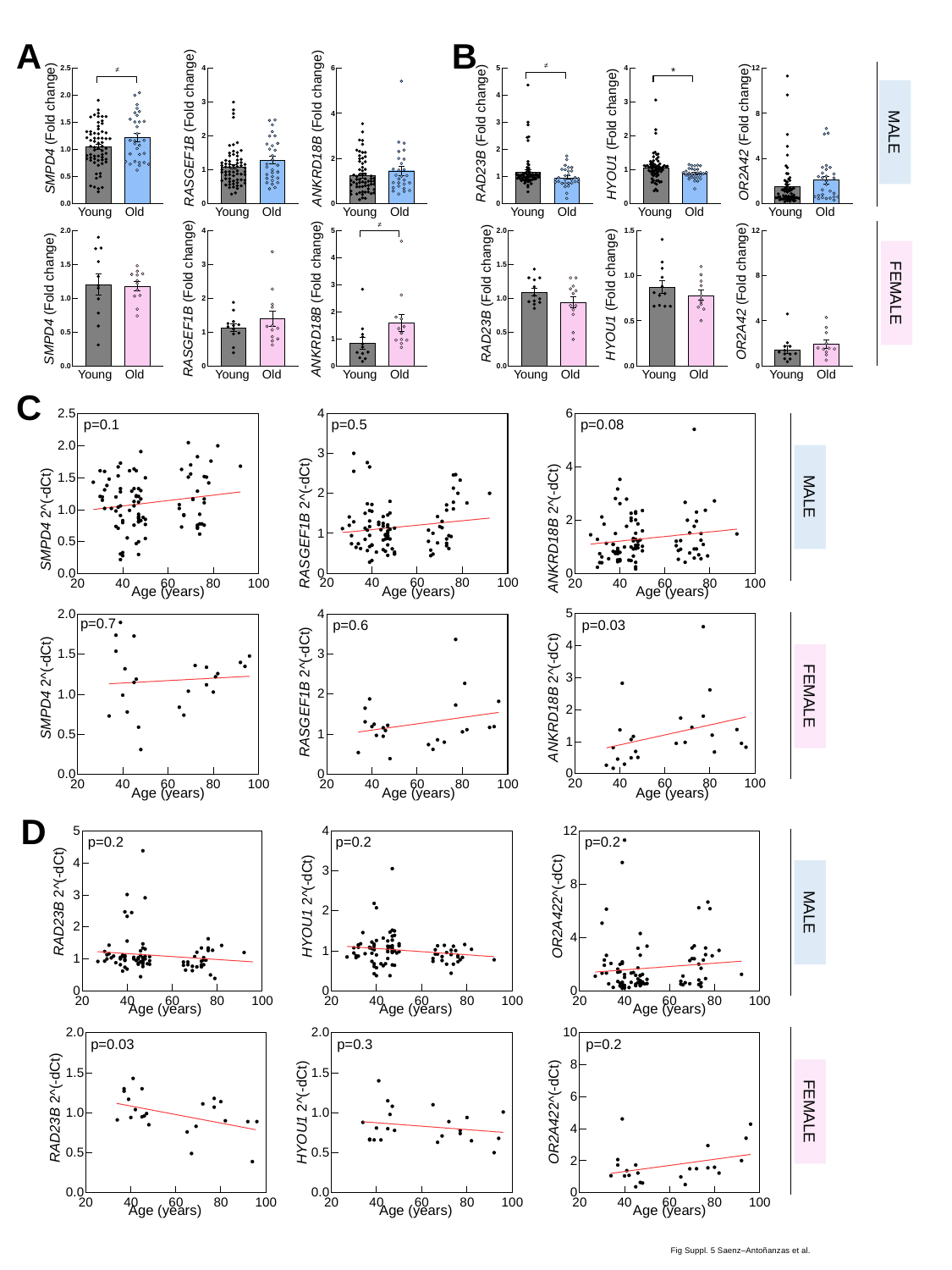

A
B
≠
*
≠
SMPD4 (Fold change)
HYOU1 (Fold change)
OR2A42 (Fold change)
ANKRD18B (Fold change)
RASGEF1B (Fold change)
RAD23B (Fold change)
MALE
Young
 Old
Young
 Old
Young
 Old
Young
 Old
Young
 Old
Young
 Old
≠
OR2A42 (Fold change)
HYOU1 (Fold change)
RAD23B (Fold change)
SMPD4 (Fold change)
ANKRD18B (Fold change)
RASGEF1B (Fold change)
FEMALE
Young
 Old
Young
 Old
Young
 Old
Young
 Old
Young
 Old
Young
 Old
C
p=0.1
p=0.5
p=0.08
SMPD4 2^(-dCt)
RASGEF1B 2^(-dCt)
ANKRD18B 2^(-dCt)
MALE
Age (years)
Age (years)
Age (years)
SMPD4 2^(-dCt)
p=0.7
p=0.03
p=0.6
RASGEF1B 2^(-dCt)
ANKRD18B 2^(-dCt)
FEMALE
Age (years)
Age (years)
Age (years)
D
RAD23B 2^(-dCt)
HYOU1 2^(-dCt)
OR2A422^(-dCt)
p=0.2
p=0.2
p=0.2
MALE
Age (years)
Age (years)
Age (years)
p=0.2
p=0.3
p=0.03
RAD23B 2^(-dCt)
HYOU1 2^(-dCt)
OR2A422^(-dCt)
FEMALE
Age (years)
Age (years)
Age (years)
Fig Suppl. 5 Saenz–Antoñanzas et al.

## Slide 6
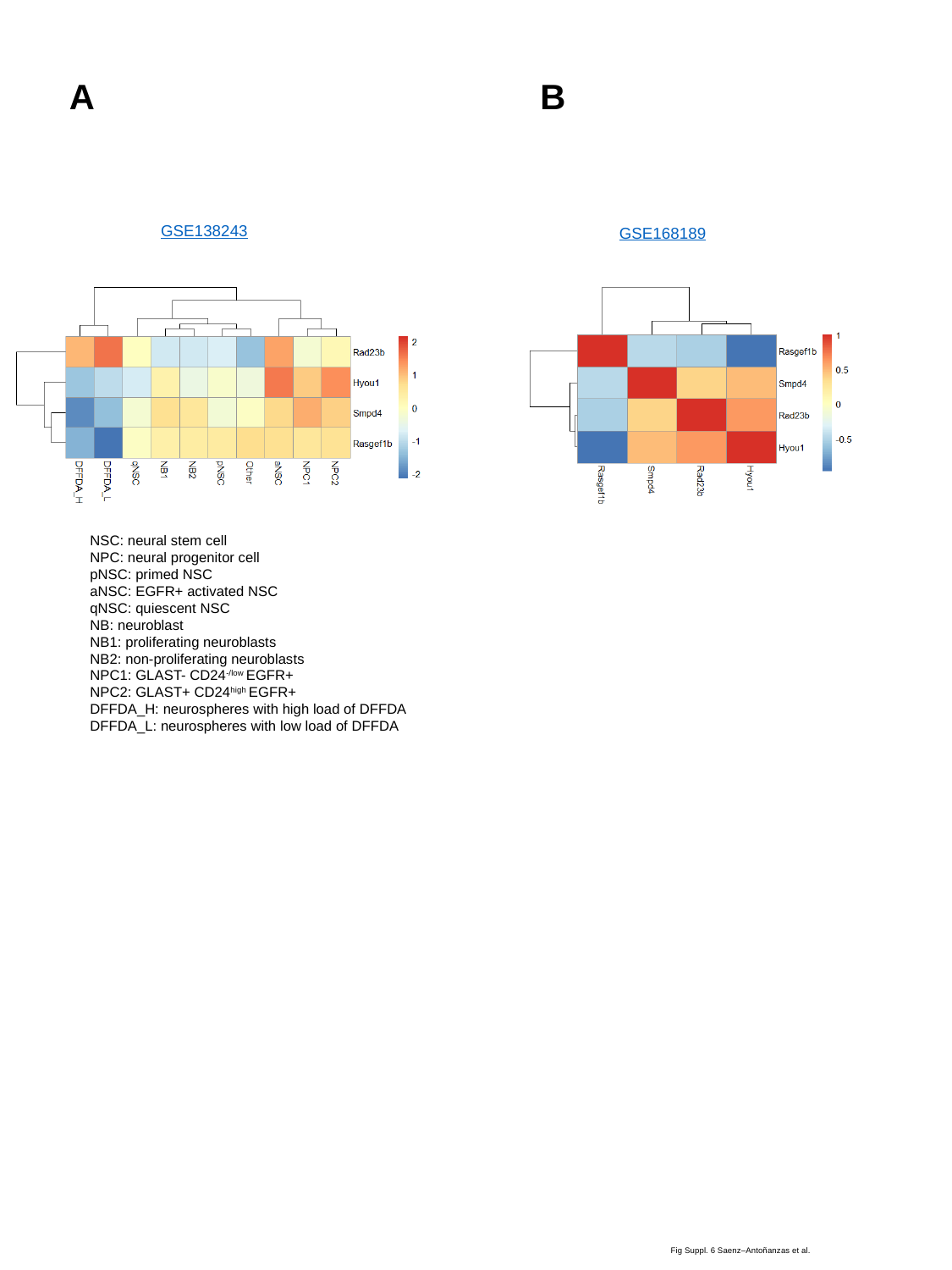

A
B
# GSE138243
GSE168189
NSC: neural stem cell
NPC: neural progenitor cell
pNSC: primed NSC
aNSC: EGFR+ activated NSC
qNSC: quiescent NSC
NB: neuroblast
NB1: proliferating neuroblasts
NB2: non-proliferating neuroblasts
NPC1: GLAST- CD24-/low EGFR+
NPC2: GLAST+ CD24high EGFR+
DFFDA_H: neurospheres with high load of DFFDA
DFFDA_L: neurospheres with low load of DFFDA
Fig Suppl. 6 Saenz–Antoñanzas et al.

## Slide 7
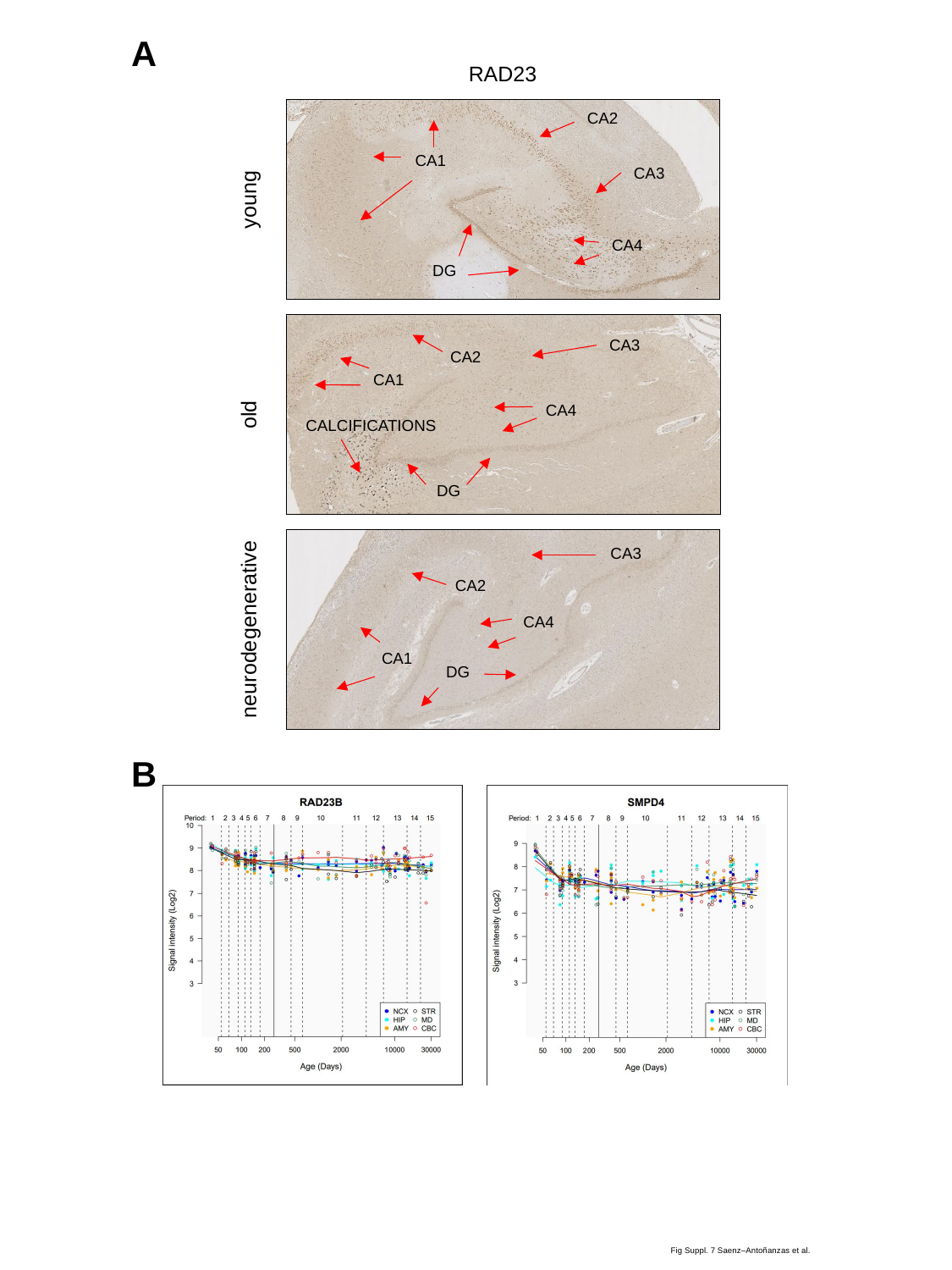

A
RAD23
CA2
CA1
CA3
CA4
DG
young
CA3
CA2
CA1
CA4
CALCIFICATIONS
DG
old
CA3
CA2
CA4
CA1
DG
neurodegenerative
B
Fig Suppl. 7 Saenz–Antoñanzas et al.

## Slide 8
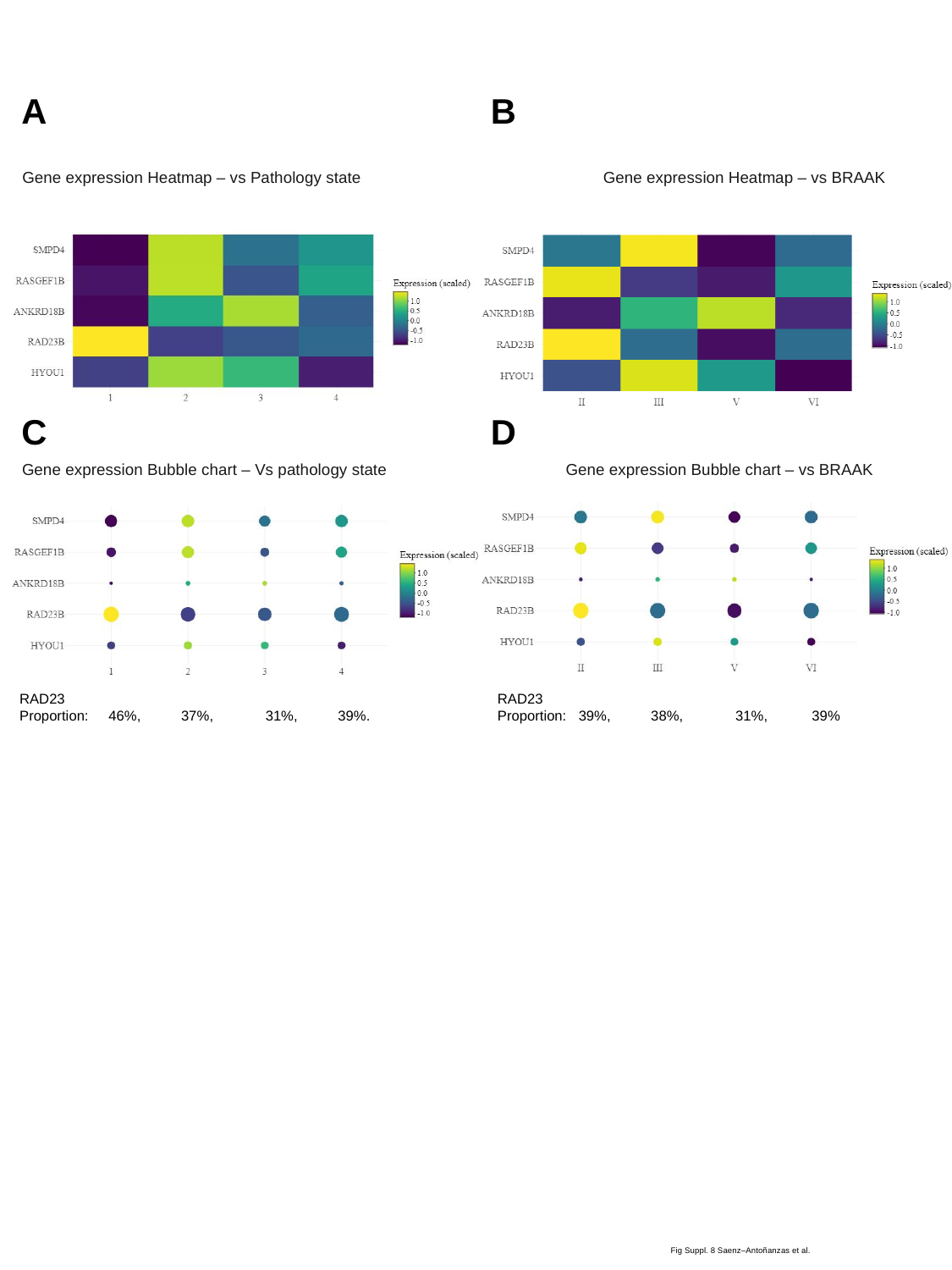

A
B
Gene expression Heatmap – vs Pathology state
Gene expression Heatmap – vs BRAAK
C
D
Gene expression Bubble chart – Vs pathology state
Gene expression Bubble chart – vs BRAAK
RAD23
Proportion: 46%, 37%, 31%, 39%.
RAD23
Proportion: 39%, 38%, 31%, 39%
Fig Suppl. 8 Saenz–Antoñanzas et al.

## Slide 9
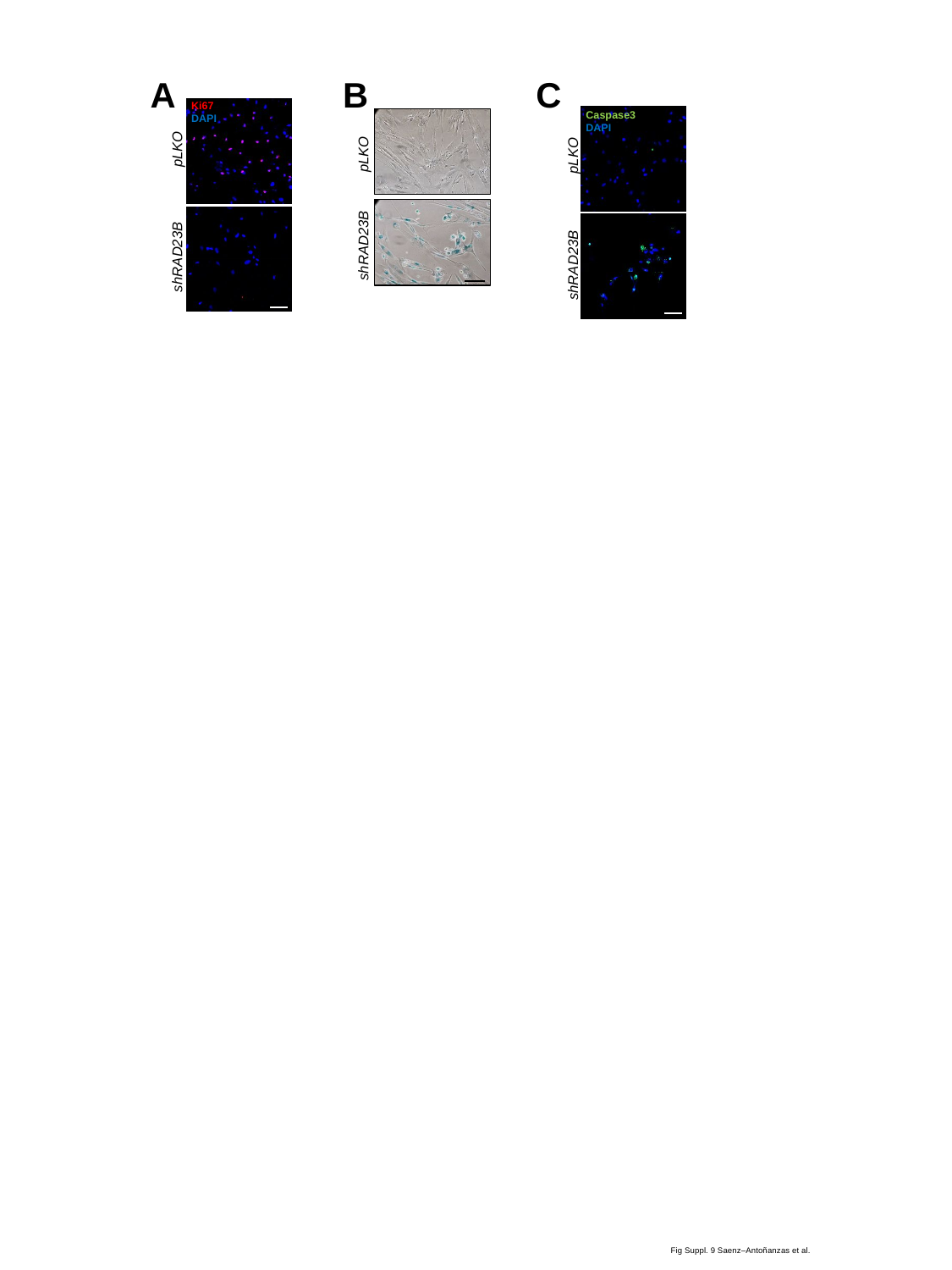

A
B
C
Ki67
DAPI
pLKO
shRAD23B
pLKO
shRAD23B
Caspase3
DAPI
pLKO
shRAD23B
Fig Suppl. 9 Saenz–Antoñanzas et al.
