# Supplementary material for: Differential Gene Expression in Human Hippocampus With Aging
Source: Aging Cell. 2026 Mar 26;25(4):e70459. doi: 10.1111/acel.70459 (PMC13140639; doi:10.1111/acel.70459)
Supplement: Supplementary file 2 — Table S1: Information of the human hippocampus samples used for the transcriptomic study (cohort 1) divided by groups. Table S2: Information of the human hippocampus samples used for mRNA validations (extension of cohort 1) divided by groups. Table S3: Information of the human hippocampus samples (cohort 2) used for protein studies (immunofluorescence and immunohistochemistry) divided by groups. [file ACEL-25-e70459-s003.docx]

**Table 1.** Information of the human hippocampus samples used for the transcriptomic study (cohort 1) divided by groups.

| Sample | Age (years) | Sex | Group | Clinical history | RIN |
| --- | --- | --- | --- | --- | --- |
| 1 | 27 | M | young | Unknown | Unknown |
| 2 | 30 | M | young | Unknown | Unknown |
| 3 | 33 | M | young | Unknown | Unknown |
| 4 | 35 | M | young | Unknown | Unknown |
| 5 | 49 | F | young | None | 4,7 |
| 6 | 58 | M | old | Mild cerebral angiopathy | 4,1 |
| 7 | 62 | M | old | None | 6,3 |
| 8 | 70 | M | old | Unknown | Unknown |
| 9 | 75 | M | old | Unknown | Unknown |
| 10 | 76 | M | old | Mild cerebral angiopathy | 4,4 |
| 11 | 77 | M | old | Unknown | Unknown |
| 12 | 79 | M | old | None | 5,8 |
| 13 | 88 | F | old | Vascular dementia | 5,4 |
| 14 | 97 | F | old | Vascular dementia | 4,8 |
| 15 | 99 | F | old | Vascular dementia | 5,4 |
| 16 | 100 | M | old | None | 7,8 |

M- Male; F- Female

**Table 2.** Information of the human hippocampus samples used for mRNA validations (extension of cohort 1) divided by groups.

| Sample | Age (years) | Sex | Group | Use (validation) | Cause of death | Clinical history |
| --- | --- | --- | --- | --- | --- | --- |
| 1 | 27 | M | young | DEGs and correlation | Anoxia | None |
| 2 | 30 | M | young | DEGs and correlation | Trauma | None |
| 3 | 30 | M | young | DEGs and correlation | Traumatic Brain Injury | None |
| 4 | 31 | M | young | DEGs and correlation | Sudden death | Unknown |
| 5 | 31 | M | young | DEGs and correlation | Trauma | Unknown |
| 6 | 32 | M | young | DEGs and correlation | Cardiac etiology | Cardiac (Cardiac arrhythmia due to arrhythmogenic dysplasia [autopsy finding]) |
| 7 | 32 | M | young | DEGs and correlation | Anoxia | Unknown |
| 8 | 32 | M | young | DEGs and correlation | Anoxia | Unknown |
| 9 | 34 | F | young | DEGs and correlation | Anoxia | None |
| 10 | 37 | F | young | DEGs and correlation | Unknown | None |
| 11 | 37 | F | young | DEGs and correlation | Toxic agents | Toxic agents |
| 12 | 39 | F | young | DEGs and correlation | Sudden death | None |
| 13 | 40 | F | young | DEGs and correlation | Anoxia | None |
| 14 | 41 | F | young | DEGs and correlation | Anoxia | Unknown |
| 15 | 42 | F | young | DEGs and correlation | Sudden death | None |
| 16 | 45 | F | young | DEGs and correlation | Traumatic Brain Injury | Neoplasm (Unspecified tumour) |
| 17 | 33 | M | young | Correlation | Suicide | Unknown |
| 18 | 34 | M | young | Correlation | Trauma | Gastrointestinal (Hepatic damage) |
| 19 | 35 | M | young | Correlation | Cardiac etiology | None |
| 20 | 36 | M | young | Correlation | Sudden death | Unknown |
| 21 | 37 | M | young | Correlation | Trauma | None |
| 22 | 37 | M | young | Correlation | Anoxia | None |
| 23 | 37 | M | young | Correlation | Sudden death | None |
| 24 | 38 | M | young | Correlation | Trauma | None |
| 25 | 38 | M | young | Correlation | Unknown | None |
| 26 | 38 | M | young | Correlation | Cardiac etiology | None |
| 27 | 38 | M | young | Correlation | Sudden death | None |
| 28 | 39 | M | young | Correlation | Anoxia | None |
| 29 | 39 | M | young | Correlation | Anoxia | None |
| 30 | 39 | M | young | Correlation | Sudden death | None |
| 31 | 39 | M | young | Correlation | Sudden death | None |
| 32 | 39 | M | young | Correlation | Toxic agents | Psychiatric (Schizophrenia and substance abuse) |
| 33 | 39 | M | young | Correlation | Sudden death | None |
| 34 | 39 | M | young | Correlation | Sudden death | Unknown |
| 35 | 39 | M | young | Correlation | Sudden death | Unknown |
| 36 | 40 | M | young | Correlation | Cardiac etiology | Cardiac (Acute ischemic heart disease) |
| 37 | 40 | M | young | Correlation | Hypovolemic shock | None |
| 38 | 40 | M | young | Correlation | Sudden death | Unknown |
| 39 | 40 | M | young | Correlation | Trauma | Unknown |
| 40 | 40 | M | young | Correlation | Anoxia | Unknown |
| 41 | 42 | M | young | Correlation | Sudden death | None |
| 42 | 43 | M | young | Correlation | Cardiac etiology | None |
| 43 | 43 | M | young | Correlation | Sudden death | Unknown |
| 44 | 44 | M | young | Correlation | Unknown | Unknown |
| 45 | 45 | M | young | Correlation | Sudden death | None |
| 46 | 45 | M | young | Correlation | Trauma | None |
| 47 | 45 | F | young | Correlation | Cerebrovascular accident | None |
| 48 | 45 | M | young | Correlation | Traumatic Brain Injury | None |
| 49 | 45 | M | young | Correlation | Trauma | Unknown |
| 50 | 45 | M | young | Correlation | Trauma | Unknown |
| 51 | 46 | M | young | Correlation | Cardiac etiology | Cardiac (Unspecified cardiac disease) |
| 52 | 46 | M | young | Correlation | Hypovolemic shock | None |
| 53 | 46 | F | young | Correlation | Sudden death | None |
| 54 | 46 | M | young | Correlation | Trauma | None |
| 55 | 46 | M | young | Correlation | Sudden death | Cardiac (Cardiac hypertrophy) |
| 56 | 47 | M | young | Correlation | Sudden death | Psychiatric (Unspecified mental or behavioural diseases) |
| 57 | 47 | M | young | Correlation | Cardiac etiology | None |
| 58 | 47 | M | young | Correlation | Sudden death | None |
| 59 | 47 | M | young | Correlation | Anoxia | Neoplasm (Pulmonary neoplasm) |
| 60 | 47 | M | young | Correlation | Traumatic Brain Injury | None |
| 61 | 47 | M | young | Correlation | Sudden death | Various (Liver disease and hepatic steatosis. Acute ischemic heart disease) |
| 62 | 47 | M | young | Correlation | Trauma | None |
| 63 | 47 | M | young | Correlation | Hypovolemic shock | None |
| 64 | 47 | M | young | Correlation | Trauma | Unknown |
| 65 | 47 | M | young | Correlation | Cardiac etiology | Unknown |
| 66 | 47 | M | young | Correlation | Sudden death | Unknown |
| 67 | 47 | F | young | Correlation | Unknown | Unknown |
| 68 | 47 | M | young | Correlation | Anoxia | Unknown |
| 69 | 48 | M | young | Correlation | Infection | Neoplasm (History of operated brain tumor and syringomyelia) |
| 70 | 48 | M | young | Correlation | Anoxia | None |
| 71 | 48 | M | young | Correlation | Sudden death | None |
| 72 | 48 | M | young | Correlation | Sudden death | None |
| 73 | 48 | F | young | Correlation | Anoxia | Unknown |
| 74 | 49 | M | young | Correlation | Sudden death | Unknown |
| 75 | 50 | M | young | Correlation | Sudden death | None |
| 76 | 50 | M | young | Correlation | Sudden death | None |
| 77 | 50 | M | young | Correlation | Sudden death | None |
| 78 | 50 | M | young | Correlation | Anoxia | None |
| 79 | 65 | M | old | Correlation | Cardiac etiology | Cardiac (Arrhythmia) |
| 80 | 65 | F | old | Correlation | Trauma | None |
| 81 | 65 | M | old | Correlation | Suicide | Unknown |
| 82 | 66 | M | old | Correlation | Anoxia | Neurologic (Parkinson’s disease with genetic mutation) |
| 83 | 66 | M | old | Correlation | Sudden death | None |
| 84 | 67 | M | old | Correlation | Unknown | None |
| 85 | 67 | F | old | Correlation | Sudden death | None |
| 86 | 67 | M | old | Correlation | Trauma | Unknown |
| 87 | 69 | F | old | Correlation | Suicide | Various (Unspecified mental or behavioral disorders and circulatory or cardiac diseases) |
| 88 | 69 | M | old | Correlation | Anoxia | Cardiac (Ischemic heart disease) |
| 89 | 69 | M | old | Correlation | Anoxia | None |
| 90 | 70 | M | old | Correlation | Traumatic Brain Injury | None |
| 91 | 70 | M | old | Correlation | Trauma | None |
| 92 | 71 | M | old | Correlation | Suicide | Psychiatric (Unspecified mental or behavioural diseases) |
| 93 | 71 | M | old | Correlation | Hypovolemic shock | Cardiac (Ischemic heart disease) |
| 94 | 72 | F | old | Correlation | Trauma | None |
| 95 | 73 | M | old | Correlation | Trauma | None |
| 96 | 73 | M | old | Correlation | Traumatic Brain Injury | None |
| 97 | 73 | M | old | Correlation | Anoxia | Various (Infectious diseases: pneumonia. Neoplasm: gastrointestinal cancer. Neurological diseases: epilepsy) |
| 98 | 73 | M | old | Correlation | Unknown | Unknown |
| 99 | 73 | M | old | Correlation | Unknown | Unknown |
| 100 | 74 | M | old | Correlation | Anoxia | None |
| 101 | 74 | M | old | Correlation | Cardiac etiology | None |
| 102 | 74 | M | old | Correlation | Traumatic Brain Injury | Unknown |
| 103 | 75 | M | old | Correlation | Trauma | Neoplasm (Unspecified tumour) |
| 104 | 76 | M | old | DEGs and correlation | Traumatic Brain Injury | None |
| 105 | 76 | M | old | DEGs and correlation | Anoxia | None |
| 106 | 76 | M | old | DEGs and correlation | Anoxia | None |
| 107 | 76 | M | old | DEGs and correlation | Sudden death | None |
| 108 | 77 | M | old | DEGs and correlation | Traumatic Brain Injury | None |
| 109 | 77 | F | old | DEGs and correlation | Anoxia | Unknown |
| 110 | 77 | F | old | DEGs and correlation | Unknown | Unknown |
| 111 | 78 | M | old | DEGs and correlation | Traumatic Brain Injury | Neoplasm (Operated colon and laryngeal neoplasms) |
| 112 | 79 | M | old | DEGs and correlation | Anoxia | None |
| 113 | 80 | F | old | DEGs and correlation | Cardiac etiology | Cardiac (Arterial or venous diseases) |
| 114 | 81 | F | old | DEGs and correlation | Traumatic Brain Injury | Cardiac (Unspecified cardiac disease) |
| 115 | 82 | M | old | DEGs and correlation | Cardiac etiology | None |
| 116 | 82 | F | old | DEGs and correlation | Unknown | Unknown |
| 117 | 92 | M | old | DEGs and correlation | Trauma | Unknown |
| 118 | 92 | F | old | DEGs and correlation | Trauma | Unknown |
| 119 | 94 | F | old | DEGs and correlation | Trauma | Unknown |
| 120 | 96 | F | old | DEGs and correlation | Unknown | Unknown |

M- Male; F- Female; DEGs- Differentially Expressed Genes

**Table 3.** Information of the human hippocampus samples (cohort 2) used for protein studies (immunofluorescence and immunohistochemistry) divided by groups.

| Sample | Age (years) | Sex | Group | Use | Cause of death | Clinical history |
| --- | --- | --- | --- | --- | --- | --- |
| 1 | 28 | M | young | IF | Unknown | None |
| 2 | 29 | M | young | IF | Unknown | ARTAG (Astrogliopathy) |
| 3 | 43 | F | young | IF | Septic shock | None |
| 4 | 36 | M | young | IHC | Unknown | Cerebral vascular lipohyalinosis (associated with diabetes). Unspecified psychiatric disorder. |
| 5 | 44 | M | young | IHC | Hepatitis | None |
| 6 | 47 | M | young | IHC | Neoplasm | Depressive disorder |
| 7 | 58 | M | old | IHC | Neoplasm | None |
| 8 | 65 | M | old | IHC | Pulmonary hemorrhage | Atrial fibrillation. Hemochromatosis. Anxiety |
| 9 | 66 | F | old | IHC | Neoplasm (Biliopancreatic adenocarcinoma) | None |
| 10 | 67 | M | old | IHC | Neoplasm (Laryngeal squamous cell carcinoma) | None |
| 11 | 77 | M | old | IHC | Unknown | Bitemporal hematoma of probable traumatic origin. Alcoholism |
| 12 | 78 | F | old | IHC | Unknown | Cerebral ischemia - Disseminated intravascular coagulation |
| 13 | 85 | M | old | IHC | Septic shock | Chronic Obstructive Pulmonary Disease. Chronic myelomonocytic leukemia type I (stable, not meeting criteria for treatment; under clinical observation). Alcoholism |
| 14 | 85 | M | old | IHC | Hypovolemia | Atral fibrillation. Heart failure. Lupus |
| 15 | 87 | M | old | IHC | Neoplasm | Chronic Obstructive Pulmonary Disease. Heart failure. |
| 16 | 74 | M | old | IF | Septic shock | Hypertension. Heart failure. Atrial fibrillation. Mitral insufficiency. Chronic Obstructive Pulmonary Disease. Renal failure. Diabetes |
| 17 | 76 | F | old | IF | Septic shock | Hypertensión arterial. Hypercholesterolemia. |
| 18 | 90 | M | old | IF | Unknown | Vascular dementia. Cerebral angiopathy. |
| 19 | 62 | M | AD | IHC | End-stage neurodegenerative disease | Urinary incontinence. Anxiety. AD BRAAK VI. Cerebral angiopathy |
| 20 | 68 | F | AD | IHC | End-stage neurodegenerative disease | Hypertension. Depressive disorder. AD BRAAK VI. Cerebral angiopathy |
| 21 | 71 | M | AD | IHC | End-stage neurodegenerative disease | Hypertension. Diabetes. AD BRAAK I . Multiple System Atrophy. |
| 22 | 72 | F | AD | IHC | End-stage neurodegenerative disease | None. AD BRAAK VI. Cerebral angiopathy |
| 23 | 78 | F | AD | IHC | End-stage neurodegenerative disease | Hypertension. Epilepsy. Renal failure. Chronic hepatitis C. AD BRAAK VI . Cerebral angiopathy |
| 24 | 79 | M | AD | IHC | End-stage neurodegenerative disease | Chronic Obstructive Pulmonary Disease. Hypercholesterolemia. Hypertension. AD BRAAK I / ALPHA-SYNUCLEIN-BRAAK VI |
| 25 | 79 | M | AD | IHC | End-stage neurodegenerative disease | Hyperlipidemia. Hypertension. Hypothyroidism. AD BRAAK II / Progressive supranuclear palsy |
| 26 | 82 | M | AD | IHC | End-stage neurodegenerative disease | Chronic Obstructive Pulmonary Disease. Psoriasis. Hypertension. Cholelithiasis. Renal tumour. AD BRAAK VI / ALPHA-SYNUCLEIN-BRAAK VI |

M- Male; F- Female; IHC- Immunohistochemistry; AD- Alzheimer disease
